# Transfer of chloroplast *rbcL* and *atpB* genes to nucleus enables partial rescue of photoautotrophic growth of Chlamydomonas

**DOI:** 10.1016/j.synbio.2026.07.004

**Published:** 2026-07-30

**Authors:** Weida Shi, Mengyue Zhu, Chunhui Hou, Qiuling Fan, Deqiang Duanmu

**Affiliations:** a State Key Laboratory of Agricultural Microbiology, College of Bio-X, Hubei Hongshan Laboratory, Huazhong Agricultural University, Wuhan, China; b College of Life Science and Technology, Huazhong Agricultural University, Wuhan, China; c Shenzhen Branch, Guangdong Laboratory for Lingnan Modern Agriculture, Genome Analysis Laboratory of the Ministry of Agriculture, Agricultural Genomics Institute at Shenzhen, Chinese Academy of Agricultural Sciences, Shenzhen, China

**Keywords:** Chloroplast genome evolution, Endosymbiosis, Gene transfer, *rbcL*, *atpB*, Minimal chloroplast genome

## Abstract

An ancient cyanobacterium has evolved through endosymbiosis to form extant chloroplasts of eukaryotic algae and higher plants. During this process, most genes have been transferred to the nuclear genomes. The chloroplast gene sets of different photosynthetic species are relatively conserved yet still exhibiting differences among different species, suggesting a dynamic process and divergence of chloroplast-to-nucleus gene transfer events. Here, based on a comparison of 15 representative green lineage species with sequenced chloroplast genomes, we selected two genes, *atpB* and *rbcL*, from a set of 41 conserved genes for chloroplast-to-nucleus gene transfer test. Using the green alga *Chlamydomonas reinhardtii* as a model organism, we expressed these two genes in the nuclear genome in the corresponding chloroplast mutant background. We demonstrate that transferring *atpB* and *rbcL* to the nucleus sustains photoautotrophic growth at different levels. The *atpB*-TN (transfer-to-nucleus) strains retained photoautotrophic growth to a substantial extent, whereas the *rbcL*-TN strains were able to grow photoautotrophically only under 5% CO_2_ and not under ambient air (∼0.04% CO_2_). The nucleus-encoded proteins accumulated to levels substantially lower than those of the chloroplast-expressed proteins, reaching only 7∼10% (for ATPB) and less than 6% (for RBCL) of the wild-type level. Although both two proteins were relocalized to the chloroplast and assembled into respective ATP synthase and Rubisco complexes, their low abundance appears to account for the incomplete restoration of photosynthetic capability. This study provides a framework for progressively transferring more chloroplast protein-coding genes to the nucleus towards achieving a minimal chloroplast genome in a green algal chassis.

## 1 Introduction

Chloroplast is a semi-autonomous organelle that exists in higher plants and eukaryotic algae, essential for photosynthesis and many metabolic pathways [1]. Chloroplasts are believed to have originated from an endosymbiotic engulfment event that occurred approximately 1.5 billion years ago [2]. Specifically, an ancestral cyanobacterium was internalized by a primitive eukaryotic cell, establishing a symbiotic association that, through extensive evolutionary processes over a billion years, gave rise to modern chloroplasts [3]. Complex endosymbiotic events, including primary, secondary, and tertiary endosymbiosis, as well as the horizontal gene transfer, have played a significant role in shaping the diversity of chloroplast genomes and the evolutionary lineages of various oxygenic phototrophs [3,4]. Nonetheless, the genetic information contained within the chloroplast genome exhibits a high degree of similarity across diverse species, encoding conserved genes associated with photosynthetic CO_2_ fixation, ATP synthesis, transcription, translation, and other essential functions. Chloroplasts contain approximately 3000 proteins, the vast majority of which, exceeding 95%, are encoded by the nuclear genome [5,6]. A portion of those genes originated from the eukaryotic host, while a significant fraction was originally encoded by the cyanobacterial genome and subsequently transferred to the nucleus during the course of endosymbiotic evolution [7,8].

The transfer of chloroplast genes to the nucleus has resulted in a dramatic reduction of the chloroplast genome size, thereby alleviating the demands for DNA replication, damage repair processes, and reducing genetic stress. Concurrently, the transfer of genes to nucleus facilitates the cell nucleus to function as the central regulatory hub by encoding genes whose protein products are targeted to chloroplast. Among photosynthetic organisms sequenced to date, the typical chloroplast genome sizes range from approximately 100 to 250 kilobases. Notably, the smallest chloroplast genome identified so far among photosynthetic organisms is that of *Ostreococcus tauri*, which is roughly 70 kilobases and encodes ∼85 genes [9,10]. This raises the question of whether *O. tauri* represents the simplest model of chloroplast endosymbiotic evolution that retains photosynthetic capacity. However, phylogenetic analyses appear to challenge this interpretation. A substantial number of nuclear genes in plants have been identified as homologous to those found in cyanobacteria and chloroplast genomes of other species [11,12]. Nuclear integrants of chloroplast DNA have been identified in over 200 plant species to date [13], e.g., a ∼131 kb fragment in the rice genome, which represents nearly the entire chloroplast genome sequence inserted into the nucleus [14]. In *Ziziphus jujuba*, the proportion of chloroplast-derived sequences constitutes nearly 1.5% of the nuclear genome [13]. The widespread integrants of chloroplast DNA contribute to genetic diversity and serve as a potential gene source, accelerating the emergence of fusion genes [13]. Even so, following the transfer of chloroplast DNA into the nucleus, these sequences often remain inactive as nonfunctional DNA fragments. They may also be identified as foreign elements and subsequently silenced through DNA methylation [15]. To assimilate into part of the nuclear genome and evolve into nuclear protein-coding genes, these chloroplast-derived sequences typically undergo multiple rounds of genetic recombination and intramolecular mutations [7].

In contrast, genetic engineering can circumvent this lengthy natural process. In previous studies, a transcriptional unit of the kanamycin resistance gene *nptII*, which can only be transcribed exclusively within the nuclear genome, was first introduced into the tobacco chloroplast genome. Through kanamycin selection, progeny exhibiting nuclear expression of the transferred gene were successfully obtained, demonstrating the occurrence of gene transfer from chloroplast to the nucleus with an estimated frequency of one nuclear transfer event per five million cells [16,17]. It was observed that some chloroplast DNA fragments were co-transferred to the nuclear genome accompanying *nptII* in the progeny, resulting in novel transcripts of hybrid genes. The transcriptional activation of those hybrid genes may arise from molecular recombination that captures promoters of other nuclear genes [18]. Additionally, some nonfunctional chloroplast DNA fragments may evolve into introns that are subsequently excised during nuclear transcription [19]. Intermolecular exon shuffling further contributes to the evolutionary development of chloroplast-targeted peptides (cTPs), facilitating protein re-localization to the chloroplast [20]. Through successive rounds of DNA shuffling, these symbiogenetic genes may attain function, thereby substituting chloroplast genes [21].

Modification of nuclear genome can facilitate the gene transfer from chloroplast to nucleus, expediting the natural evolution and laying a foundation for engineering the chloroplast organelle. Here, we selected two conserved genes in chloroplast genomes, *atpB* and *rbcL*, for the transfer-to-nucleus (TN) test in the model green alga *Chlamydomonas reinhardtii*. Notably, *atpB*-TN and *rbcL*-TN can partially restore growth and photosynthetic function in their respective chloroplast gene deletion mutants but with different outcomes: *atpB* is comparatively transferable, whereas protein product of nuclear *rbcL* can reach complex assembly but still encounters a critical expression/accumulation barrier that prevents complete functional replacement of the chloroplast *rbcL* gene. Our study has demonstrated the feasibility of engineering of chloroplast gene transfer to nucleus in an algal chassis, offering a guidance for the chloroplast genome engineering and minimization.

## 2 Materials and methods

### 2.1 Chlamydomonas strains and growth conditions

*Chlamydomonas reinhardtii* strain 4A+ (CC4051 mt+, a 137c derivative), a commonly used wild-type strain, has been previously described [22]. Mutant strains, including Δ*rbcL* (CC-4696 *rbcL*Δ-MX3312 mt+) and Δ*atpB* (CC-373 ac-u-c-2-21, *atpB* deletion, mt+), were obtained from the Chlamydomonas Resource Center (https://www.chlamycollection.org/). The deletion sites of both mutants were confirmed by PCR analyses. Primer details were provided in the Supplemental Data. 4A+ was maintained on solid TAP (Tris-Acetate-Phosphate) medium at 24°C under a periodic dark/light cycle (cool white light illumination, ∼10 μmol photons m^−2^ s^−1^), whereas mutant strains were maintained in continuous darkness. Liquid cultures were grown on a shaker either mixotrophically (TAP) or photoautotrophically (TP, TAP without acetate) under continuous white light illumination (∼30 μmol photons m^−2^ s^−1^). To evaluate photosynthetic growth phenotypes, cells were cultured to logarithmic phase (∼1 × 10^6^ cells/mL), collected, washed with TP medium, and then equal numbers (∼900) of cells were spotted onto TAP or TP solid plates. Plates were incubated under various white light intensities (∼15, ∼30, or ∼160 μmol photons m^−2^ s^−1^) and CO_2_ concentrations (ambient air, ∼0.04% CO_2_; or 5% CO_2_) for ∼1 week (TAP) or ∼2 weeks (TP).

### 2.2 Plasmid construction

To facilitate nuclear expression of genes, the coding sequences of *C. reinhardtii atpB* and *rbcL* were optimized based on the nuclear codon usage preference. To facilitate chloroplast targeting of the expressed proteins, the endogenous RBCS2 chloroplast transit peptide (cTP) sequence was fused to the N-terminus of ATPB or RBCL protein. Additionally, multiple copies of *RBCS2* intron 1 were inserted into the optimized sequence, following an approach described before [23], to enhance gene transcriptional efficiency and pre-mRNA splicing. The optimized and modified sequences were synthesized by GenScript (Nanjing, China) and cloned into the hygromycin-resistant pHyg3 vector [24], generating pHyg3-ATPB and pHyg3-RBCL, respectively. Prior to transformation, the pHyg3-ATPB and pHyg3-RBCL plasmids were linearized by double digestion with *Not*I and *Kpn*I restriction endonucleases. The resulting linear fragments containing expression cassettes for the target genes were gel-purified and used for nuclear transformation of Chlamydomonas Δ*rbcL* or Δ*atpB* mutant cells.

### 2.3 Nuclear electroporation of Chlamydomonas cells

The Chlamydomonas cells were cultured in TAP liquid medium until reaching the logarithmic growth phase (∼1 × 10^6^ cells/mL). Cells were then harvested and suspended in TAP liquid medium supplemented with 60 mM sucrose to a final concentration of ∼1 × 10^8^ cells/mL. For each electroporation cuvette (4 mm gap), 250 μL of the cell suspension along with 100-200 ng of DNA was added, and the cuvettes were placed into a twin wave electroporator (BTX Gemini X2). Electroporation was performed using the following parameters: voltage of 800 V, resistance of 1575 Ω, and capacitance of 50 μF. Following electroporation, Chlamydomonas cells were allowed to recover in 60 mM TAP liquid medium under dark conditions for 24 h. Subsequently, they were plated onto TAP and TP solid media supplemented with 10 μg/mL of hygromycin B (Sigma #H3274) and cultured under varying intensities of white light (∼30, or ∼400 μmol photons m^−2^ s^−1^) for 4-7 days to allow viable transformants to appear.

### 2.4 Chloroplast biolistic transformation of Chlamydomonas cells

Chloroplast transformation was performed using a Bio-Rad Biolistic PDS-1000/He system. Briefly, Cells from 300 mL cultures were harvested, resuspended, and spotted onto TAP plates to form a lawn approximately the size of a golf ball prior to bombardment. Approximately 5 μg of plasmid DNA was added to 1.5 mg of aliquoted gold microcarriers (Bio-Rad #1652262), mixed with 25 μL of 2.5 M CaCl_2_ and 10 μL of 0.1 M spermidine (Sigma #S0266). The mixture was then vortexed thoroughly and centrifuged at 13000 *g* for 2 min. Pellets were washed with pure ethanol and finally resuspended in 30 μL pure ethanol. The DNA-coated gold particles were bombarded into Chlamydomonas chloroplasts using 1350 psi rupture disks (Bio-Rad #1652330). Following bombardment, cells were plated onto TP agar plates for selection of genetically complemented strains. Putative transformants were verified by PCR analysis, and positive colonies were subjected to two to three rounds of repeated single-colony selection on TP medium to achieve homoplasmy of the introduced chloroplast gene expression cassettes.

### 2.5 Immunoblot analyses

Chlamydomonas total proteins were extracted using methanol and chloroform based on the method described in a previous study [22]. Total proteins were dissolved in sample buffer (50 mM Tris-HCl, 5% (w/v) SDS) and quantified using a BCA protein assay kit (Sangon Biotech, Shanghai, China; #C503021). Subsequently, proteins were separated by SDS-PAGE and transferred onto a PVDF membrane. Following blocking with 5% skim milk, the membrane was incubated sequentially with primary antibodies (diluted 1:1000) and then HRP-conjugated goat anti-rabbit IgG (diluted 1:5000; CWBio, CW0103S). Most primary antibodies used in this study were from PhytoAB, including ATPA (PHY5699S), ATPB (PHY0312), CP43 (PHY5661S), CP47 (PHY5652S), D1 (PHY5707A), H3 (PHY2460A), LHCA1 (PHY5616S), LHCA3 (PHY5609S), LHCA4 (PHY5606S), LHCA6 (PHY5619S), LHCA8 (PHY5620S), LHCBM2/7 (PHY5669S), LHCBM5 (PHY5667S), PsaD (PHY5628S), PetB (PHY5698S), RBCL (PHY0096A), and RBCS (PHY5695A). The D2 and PsaA antibodies were generous gifts from Professor Jean-David Rochaix (University of Geneva). Immunoblot signals were detected using an enhanced chemiluminescence kit (Bio-Rad, 170-5060) and observed by a chemiluminescence imaging system (Tanon, Shanghai, China; 4600 Multi).

### 2.6 Extraction of native protein and CN-PAGE

Clear-native PAGE (CN-PAGE) was performed following a modified protocol as described previously [25]. Briefly, 150 mL Chlamydomonas cultures were collected and resuspended in the protein extraction buffer (50 mM Tris-HCl pH 8.0, 10 mM MgCl_2_, 5 mM dithiothreitol (DTT), 1 mM PMSF, 0.5% triton X-100, and 1% (v/v) cocktail protease inhibitor). Cells were broken using a high-pressure homogenizer at 8000 psi and placed on ice for 20 min to allow the membrane proteins to dissolve sufficiently. Cell debris and unbroken cells were removed by centrifugation at 4000*g* for 10 min at 4°C. The supernatant fractions were collected and mixed with the nondenatured sample buffer (80% (v/v) glycerol, 0.05% (w/v) bromophenol blue). Samples of equal chlorophyll contents were loaded onto 4-20% gradient gels. For chlorophyll measurement, 50 μL of native extract was mixed with 950 μL of DMF (dimethylformamide) and vortexed thoroughly. After centrifugation at 10000*g* for 5 min at 4°C, the supernatant was used for absorbance measurement and Chlorophyll content was calculated using the following formula: total Chl = 17.67 × A_646_._8_ + 7.12 × A_663_._8_ [26]. Electrophoresis was performed at 4°C, beginning at 50 V and increasing by 25 V every 30 min until the bromophenol blue tracking dye reached the bottom of the gel. For immunoblotting, the native gels were first incubated in denaturing buffer (2% SDS, 0.67% β-Mercaptoethanol) for 1 h, then transferred to PVDF membranes. The remaining immunoblotting steps were the same as described above.

### 2.7 Thylakoid protein purification and BN-PAGE

Thylakoid membrane isolation and blue native polyacrylamide gel electrophoresis (BN-PAGE) were performed as previously described [27]. Briefly, 500 mL of Chlamydomonas cultures were harvested and cell pellets were resuspended in ice-cold HEPES buffer (25 mM HEPES, 5 mM MgCl_2_, 0.3 M sucrose, 1 mM PMSF, pH 7.8). Cells were lysed through high-pressure homogenizing, and unbroken cells were removed by centrifugation at 4000 *g* for 10 min at 4°C. The supernatants were then centrifuged at 20,000 *g* for 30 min at 4°C to pellet the thylakoid membranes. The isolated thylakoid membranes were solubilized in 2% (w/v) n-dodecyl β-D-maltoside (β-DM; Sangon Biotech, Shanghai, China; #A610424) on ice for 30 min. Samples were loaded based on equal chlorophyll contents. BN-PAGE was performed using 4–20% gradient gels with a 4% stacking gel. Electrophoresis was carried out at 4°C in the dark and continued until the protein complexes were adequately separated.

### 2.8 Protein subcellular localization by immunofluorescence microscopy

Chlamydomonas cells in the early logarithmic growth phase were harvested, washed, and resuspended with PBS (phosphate-buffered saline), and then spotted onto poly-l-Lysine-coated glass slides and air-dried at room temperature for 10 min. Cells on slides were then fixed with 4% (w/v) formaldehyde in PBS for 20 min, followed by treatment with pre-cooled 100% methanol for 20 min at −20°C. The samples were then rehydrated by immersion in PBS and washed three times. Fixed cells were blocked with 1% (w/v) BSA (bovine serum albumin) for 1 h at room temperature and incubated with either anti-ATPB or anti-RBCL antibodies (diluted at 1:500) for 1 h at room temperature. After washing with PBS-T (PBS containing 0.1% Tween-20), cells were incubated with Alexa Fluor 488-conjugated goat anti-rabbit IgG antibody (Beyotime Biotechnology, Shanghai, China; 1:500 dilution) for 1 h, and then washed six times with PBS-T. For DNA visualization, cells were stained with DAPI (4′,6-diamidino-2-phenylindole; Yeasen Biotechnology, Shanghai, China; #40728ES03) at a concentration of 10 μg/mL for 10 min. After washing, antifade Mounting Medium was added, and slides were covered with high-transparency coverslips. Fluorescence images were obtained using a structured illumination super-resolution microscope (SIM, Nikon N-SIM).

### 2.9 Chlorophyll fluorescence measurement of PSII

*C. reinhardtii* cells were cultured mixotrophically to logarithmic growth phase. Prior to measurements, cells were collected, resuspended in fresh TAP medium to a chlorophyll concentration of 15 μg/mL, and then dark-acclimated for 10–30 min with gentle shaking. Chlorophyll fluorescence parameters of PSII were measured using a Dual-PAM 100 fluorometer (Walz, Germany). All instrument settings followed the standard operating procedure for light curve acquisition. For each light intensity step, cells were acclimated for 60 s to achieve steady-state photosynthesis, after which a saturating pulse was applied to record fluorescence parameters. The effective quantum yield of PSII (ΦPSII) was calculated as ΦPSII = (Fm' – Ft)/Fm', where Fm' represents the maximum fluorescence in the light-adapted state and Ft represents the steady-state fluorescence immediately prior to the saturating pulse. The redox state of the plastoquinone pool (PQ-redox) was calculated as 1 – qL = 1 – [(Fm' – Ft)/(Fm' – Fo')] × (Fo'/Ft), where Fo' is the minimal fluorescence in the light-adapted state.

### 2.10 Chloroplast genome analysis

The chloroplast genome sequences and annotations from 15 selected green lineage species were downloaded from NCBI (National Center for Biotechnology Information). Corresponding accession numbers were provided in Supplemental Table S1. We obtained the species phylogeny shown in Fig. 1 from the OneZoom database [28] by selecting only the phototrophs depicted in Fig. 1. All protein-coding genes from the selected 15 species form a pan-genome of 102 genes (Supplemental Table S1), among which 41 genes are retained in all chloroplast genomes and are defined as the conserved gene set.

**Fig. 1:**
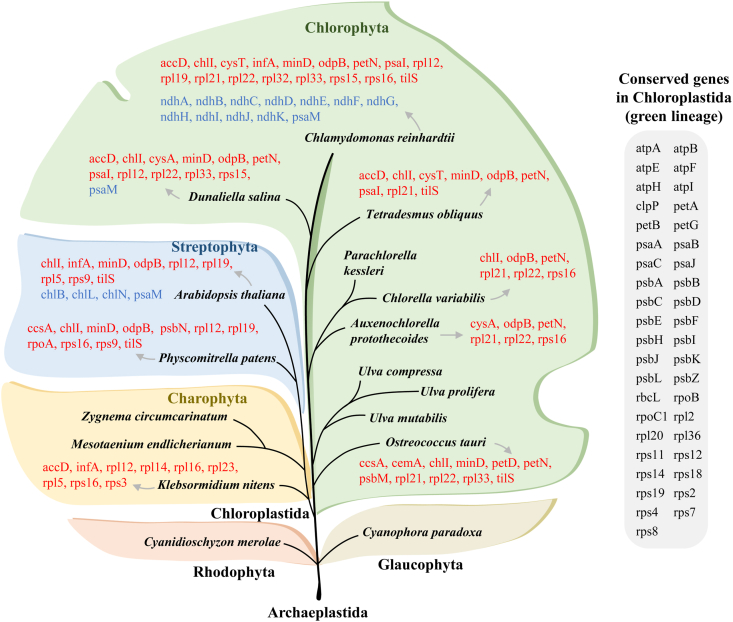
Phylogenetic distribution of gene transfers and gene losses in chloroplast genomes of representative photosynthetic species. A core set of 41 highly conserved genes is consistently retained in the plastid genomes of all analyzed 15 species of the Chloroplastida (the green lineage, gene names are listed on the right), including Charophyta, Streptophyta, and Chlorophyta. The remaining genes have either been lost from both plastid and nuclear genomes, or transferred to the nucleus in certain species, or remained unknown. Red, genes that have been transferred to the nuclear genome; Blue, genes that have been lost from both the plastid and the nuclear genomes.

Genes absent from the chloroplast genome of a given species were further analyzed to assess their distribution of “gene transfer” versus “gene loss” versus “Unknown”. Using the homologous protein sequences of the cyanobacterium *Gloeomargarita lithophora*, the closest known relative of the ancestral endosymbiotic cyanobacterium [29], *Chlamydomonas reinhardtii* (a green alga), and *Arabidopsis thaliana* (a seed plant) as queries, we performed BLAST (Basic Local Alignment Search Tool) searches against NCBI, Phytozome, and UniProt databases. The presence of nucleus-encoded homologous proteins (typically more than 30% identity over the entire lengths) with identical gene names or functional annotations suggests that the corresponding chloroplast genes may have been transferred to the nucleus and are defined as “gene transfer” (marked in red in Fig. 1 and Supplemental Table S1, the corresponding nuclear loci were listed in Supplemental Table S2). By contrast, genes that lack homologs and have also been clearly reported as evolutionary losses in previous studies are defined as “gene loss” (marked in blue in Fig. 1 and Supplemental Table S1). However, the status (transfer or loss) remains uncertain for many genes that either (i) have homologs in the corresponding nuclear genome but lacking functional annotations, or (ii) have no detectable homologs but no report of the gene loss. We took a conservative measure by defining those genes as “Unknown” (marked in grey in Supplemental Table S1).

## 3 Results

### 3.1 Conservation and variation of chloroplast genes in the green lineage

Among the eukaryotic phototrophs, the red lineage typically possesses over 200 genes [30], whereas the green lineage contains 80–130 genes [9] in their chloroplast genomes. To evaluate the potential for chloroplast-to-nucleus gene transfer, we selected 15 representative species from the green lineage with sequenced and annotated nuclear and chloroplast genomes, including members of Charophyta, Streptophyta, and Chlorophyta. We first compared their chloroplast-encoded genes and identified 41 genes that are universally conserved and consistently retained in chloroplast genomes of all analyzed species. Further BLAST analysis of respective nuclear genomes revealed that, apart from the 41 conserved genes in chloroplast genomes, some of the remaining genes have been either lost from both chloroplast and nucleus (defined as “gene loss”, marked in blue in Fig. 1 and Supplemental Table S1) or transferred to the nucleus (defined as “gene transfer”, marked in red in Fig. 1 and Supplemental Table S1, corresponding gene loci are listed in Supplemental Table S2) in at least one or another species, and the rest of the remaining genes were defined as “unknown” (marked in grey in Supplemental Table S1). As an example of “gene loss”, chloroplast-encoded NADH dehydrogenase subunit genes are retained in Charophyta and Streptophyta but are absent from *Chlamydomonas reinhardtii,* a representative Chlorophyta alga. This loss is consistent with the dispensability of these genes under temperate aquatic conditions [31]. In *C. reinhardtii*, the conventional multi-subunit complex is replaced by NDA2, a single-subunit type II NADH dehydrogenase encoded by the nuclear genome [32].

### 3.2 Construction of Chlamydomonas *atpB* and *rbcL* transfer-to-nucleus (TN) strains

We then attempted to test whether those retained chloroplast genes could be transferred to the nucleus. As a proof of concept, two essential photosynthetic genes from the set of 41 conserved chloroplast genes, *atpB* and *rbcL*, respectively encoding a subunit of chloroplast ATP synthase and the large subunit of Rubisco, the primary enzyme for CO_2_ fixation during photosynthesis, were selected for gene transfer test in the model green alga *C. reinhardtii*. Chloroplast gene deletion mutant strains were employed as recipient cells, e.g., the Δ*atpB* mutant with a 2.5-kilobase (kb) deletion spanning the *atpB* gene, and the Δ*rbcL* mutant, in which the *rbcL* gene is replaced by the *aadA* cassette conferring resistance to the aminoglycosides spectinomycin (Supplemental Fig. S1a). Nuclear expression plasmids were constructed in which the coding sequences, fused to the chloroplast transit peptide (cTP) of Rubisco small subunit 2 (RBCS2), were regulated by the HSP70A/RBCS2 chimeric promoter. Three introns were incorporated into the expression cassette to enhance expression (Fig. 2a).

**Fig. 2:**
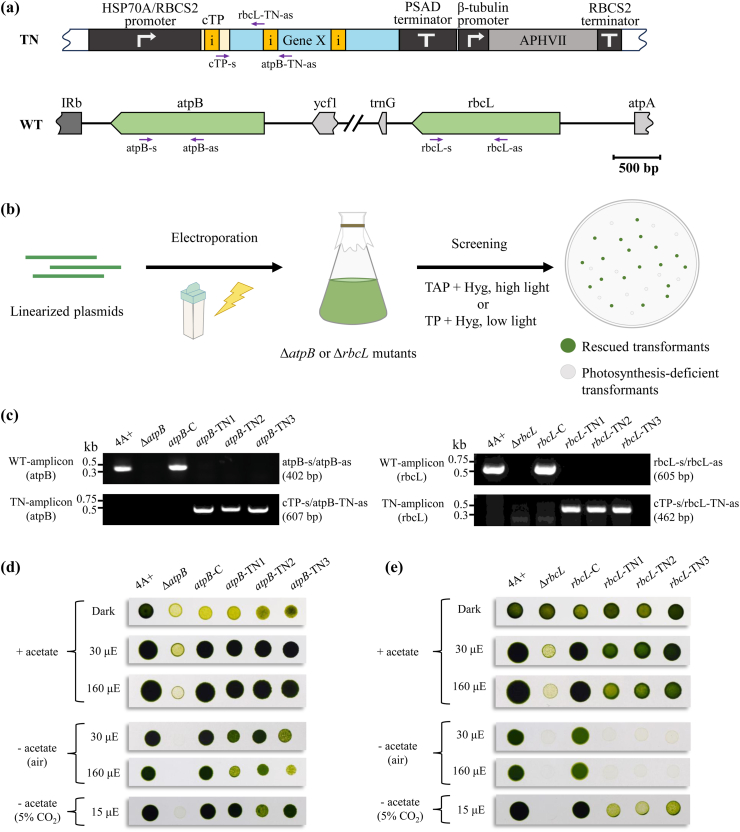
Construction and growth comparison of *Chlamydomonas reinhardtii* nuclear transformants expressing chloroplast *atpB* and *rbcL* genes. (a) Illustration of complementary plasmids for expressing Chlamydomonas chloroplast proteins in the nuclear genome. TN, transfer-to-nucleus; WT, illustration of the *atpB* and *rbcL* genes in the wild-type chloroplast genome. cTP, chloroplast transit peptide of *C. reinhardtii* Rubisco small subunit 2 (RBCS2); Gene X, nuclear codon-optimized *atpB* or *rbcL* genes; i, intron 1 of *RBCS2* gene. Names and positions of the genotyping primers are annotated. Scale bar, 500 bp. (b) Transformation and selection pipeline used in this study. After transformation, cells were plated on TAP + Hyg under high light (∼400 μmol photons m^−2^ s^−1^) or TP + Hyg under low light (∼30 μmol photons m^−2^ s^−1^), conditions under which Δ*atpB* and Δ*rbcL* mutants cannot grow. Hyg, hygromycin. (c) PCR verification of transgenic Chlamydomonas strains. WT-amplicons, PCR products amplified with primers specific to the wild-type chloroplast *atpB* or *rbcL* genes; TN-amplicons, PCR products amplified with primers specific to the nuclear codon-optimized *atpB* or *rbcL* sequences. 4A+, wild-type strain; *atpB*-C or *rbcL*-C, genetically complemented strains in Δ*atpB* or Δ*rbcL* mutants by introducing wild-type genes into the chloroplast genome; *atpB*-TN or *rbcL*-TN, nuclear transformants of Δ*atpB* or Δ*rbcL* mutants. Three independent transgenic strains were selected for *atpB*-TN or *rbcL*-TN. (d, e) Comparative growth of Chlamydomonas strains in the presence or absence of acetate under different light conditions. Air was supplemented with 5% CO_2_ for comparison. Spot assays were repeated three times and similar results were observed.

By screening transgenic strains under photoautotrophic (low light, ∼30 μmol photons m^−2^ s^−1^, no acetate) or photoheterotrophic (high light, >400 μmol photons m^−2^ s^−1^, with acetate) conditions, survival Chlamydomonas cells were obtained from both Δ*atpB* and Δ*rbcL* recipient cells, suggesting the restoration of their photosynthetic ability (Fig. 2b). These progenies were named *atpB*-TN and *rbcL*-TN, representing *atpB* or *rbcL* gene-to-nucleus transferred strains. All transgenic strains were verified by PCR detection of the wild-type (WT) or the transfer-to-nucleus (TN) amplicons (Fig. 2c). As controls, genetically complemented strains *atpB*-C and *rbcL*-C were generated via chloroplast transformation (Supplemental Fig. S1a–d).

### 3.3 Phenotypic rescue of the chloroplast Δ*atpB* and Δ*rbcL* mutants via nucleus transgenes

To characterize the phenotype of TN strains, we performed spot assays of *atpB*-TNs and *rbcL*-TNs under autotrophic (no acetate) and mixotrophic (with acetate) conditions (Fig. 2d and e). Both Δ*atpB* and Δ*rbcL* mutants were light-sensitive, exhibiting severely inhibited growth under mixotrophic conditions and no growth under photoautotrophic conditions. These deficient phenotypes can be fully complemented by native chloroplast *atpB* or *rbcL* genes (*atpB*-C or *rbcL*-C), while partially rescued by nuclear expression of respective codon-optimized genes (*atpB*-TN1/2/3 or *rbcL*-TN1/2/3). With acetate supplementation, growth of *atpB*-TNs was indistinguishable from those of the wild type 4A+ and *atpB*-C, and *atpB*-TNs showed slightly reduced photoautotrophic growth in the absence of acetate (Fig. 2d and Supplemental Fig. S2a). In contrast, transfer of *rbcL* to the nucleus only partially restored the phenotype of the Δ*rbcL* mutant, as *rbcL*-TNs grew normally under mixotrophic conditions and were capable of photoautotrophic growth when supplemented with 5% CO_2_ (Fig. 2e and Supplemental Fig. S2b). However, the photoautotrophic growth of *rbcL*-TNs were completely inhibited in ambient air (∼0.04% CO_2_, Fig. 2e). This growth defect is likely due to enhanced photorespiration and ROS (reactive oxygen species) damage, as described for the Rubisco-less mutant strains [33]. In conclusion, these results indicate that transfer of chloroplast *atpB* or *rbcL* genes to the nucleus can physiologically substitute for native chloroplast genes to various degrees.

### 3.4 ATP synthase and Rubisco assembly in the transfer-to-nucleus (TN) strains

We next compared the accumulation levels of ATPB and RBCL proteins in TN strains by immunoblotting. The accumulation of nucleus-expressed ATPB protein was markedly reduced in *atpB*-TN strains than in the chloroplast-complemented strain *atpB*-C or the wild-type 4A+. Using a dilution series of 4A+ total protein for quantification by ImageJ, the nucleus-expressed ATPB protein levels in *atpB*-TN1, *atpB*-TN2, and *atpB*-TN3 were estimated at ∼10.2%, ∼6.5%, and ∼9.5% of the 4A+ level, respectively (Fig. 3a). These transformants also accumulated a higher molecular-weight isoform of ATPB, not present in 4A+ and *atpB*-C, which likely corresponds to the full-length cTP-ATPB fusion protein. Previous evidence suggests that deficiency of the β subunit affects the synthesis and stability of other CF1 subunits and disrupts ATP synthase complex assembly [34]. Consistent with this, we found that the ATP synthase α subunit (ATPA) was absent in the Δ*atpB* mutant and its abundance was slightly recovered in *atpB*-TN strains (Fig. 3a). Likewise, the nucleus-expressed RBCL protein level in *rbcL*-TN strains was also reduced dramatically, reaching only ∼6% in *rbcL*-TN1, ∼4.5% in *rbcL*-TN2, and ∼4.3% in *rbcL*-TN3 compared to 4A+ (Fig. 3b, Supplemental Fig. S3). Correspondingly, the abundance of the nucleus-encoded Rubisco small subunit (RBCS) was also drastically reduced in those transformants (Fig. 3b). Those data suggest that subunits of the ATP synthase and Rubisco are coordinately regulated at the protein level.

**Fig. 3:**
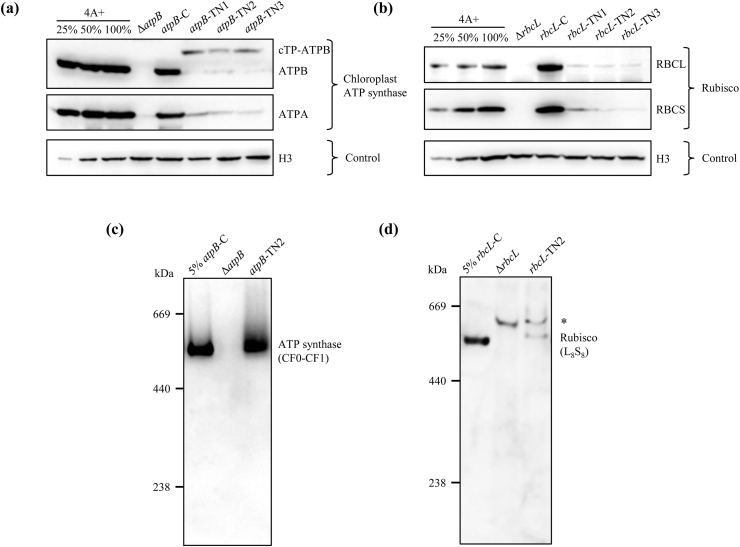
ATP synthase and Rubisco assembly in chloroplast-to-nucleus gene transfer strains. (a, b) Immunoblotting analyses of ATP synthase (a) and Rubisco (b) subunits. The higher-molecular-weight band in (a) corresponds to the uncleaved cTP-ATPB fusion protein. Similar amount of total protein (∼30 μg) was loaded for each sample. A dilution series (25% and 50%) of the 4A+ strain is used for comparison and Histone H3 was used as a loading control. (c, d) CN-PAGE analysis of ATP synthase (c) and Rubisco (d) complexes. Native proteins were extracted from the indicated strains. For mutants (Δ*atpB*, Δ*rbcL*) and gene transfer strains (*atpB*-TN2, *rbcL*-TN2), samples containing similar amounts of chlorophyll were loaded. For chloroplast-complemented strains (*atpB*-C, *rbcL*-C), a 5% dilution was used to allow direct comparison. The asterisk indicates a non-specific signal in (d).

To investigate whether the nucleus-expressed ATPB and RBCL were correctly assembled into the ATP synthase (CF0-CF1) and Rubisco holoenzymes, we performed clear-native polyacrylamide gel electrophoresis (CN-PAGE) experiments followed by immunological detection of ATPB and RBCL proteins. Immunoblotting against ATPB or RBCL detected the CF0-CF1 and Rubisco L_8_S_8_ complexes in *atpB*-TN2 and *rbcL*-TN2, without apparent monomers or assembly intermediates being observed. Contents of those two holoenzymes were much lower compared to the chloroplast-complemented strains (Fig. 3c and d), consistent with the observed low levels of subunit accumulation in SDS-PAGE and immunoblot analyses (Fig. 3a and b). These results indicate that protein products of the nucleus-encoded genes were successfully assembled into their respective complexes. The dramatically reduced levels of holoenzyme accumulation appear to be associated with the low abundance of nucleus-expressed ATPB and RBCL proteins, rather than with defects in their assembly processes.

### 3.5 Subcellular localization of nucleus-expressed ATPB and RBCL proteins

In all three *atpB*-TN strains, majority of the ATPB protein appeared to retain the cTP (Fig. 3a). In contrast, the cTP of nucleus-expressed RBCL was completely cleaved, as evidenced by the identical molecular weight of RBCL proteins in *rbcL*-TN1/2/3 and *rbcL*-C strains (Fig. 3b). To assess the efficiency of protein re-localization to the chloroplast, we examined the subcellular localization of nucleus-expressed ATPB and RBCL proteins using high-resolution structured illumination microscopy (SIM). Strains were first cultured in TAP medium and then transferred to TP medium to allow photoautotrophic growth for 24 h. Immunofluorescence revealed that ATPB and RBCL were correctly localized to the chloroplast in respective *atpB*-TN2 and *rbcL*-TN2 strains. Notably, they displayed different localization patterns within the chloroplast as expected. In *atpB*-TN2, the ATPB protein formed punctate aggregates in the chloroplast, similar to those observed in the *atpB*-C strain (Fig. 4a). Very few signals were detected in the cytoplasm, suggesting that both ATPB isoforms were imported into the chloroplast and subsequently assembled into functional ATP synthase complexes (Fig. 3c). In *rbcL*-TN2, the RBCL protein was primarily localized to the pyrenoid, similar to that observed in *rbcL*-C (Fig. 4b). Since the pyrenoid is an essential component of the CO_2_ concentrating mechanism of eukaryotic algae under limiting CO_2_ conditions [35,36], these results indicate that the first step of photosynthetic CO_2_ fixation is also mainly occurring in the pyrenoid of *rbcL*-TNs.

**Fig. 4:**
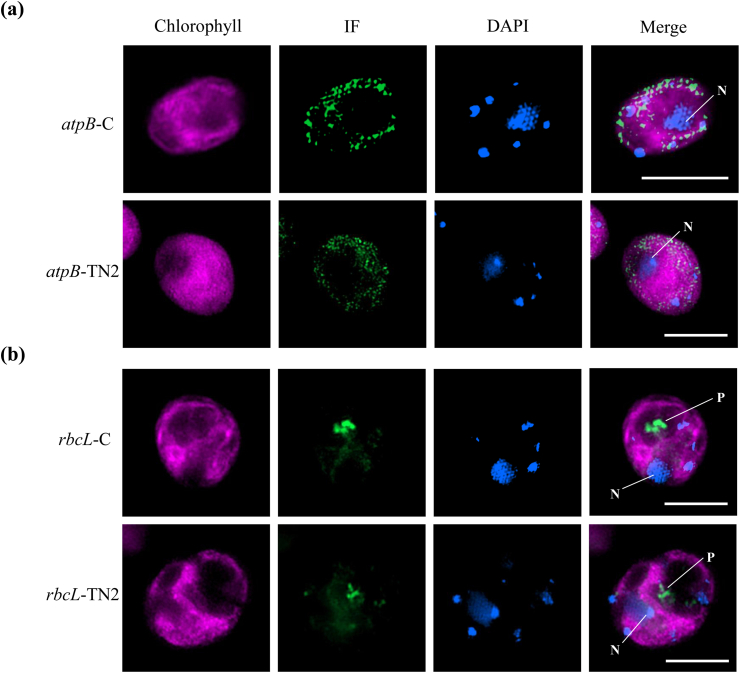
Subcellular localization of ATPB and RBCL proteins. (a) ATPB localization in the chloroplast-complemented (*atpB*-C, upper) and nucleus-transferred strains (*atpB*-TN2, lower). (b) RBCL localization in the chloroplast-complemented (*rbcL*-C, upper) and nucleus-transferred strains (*rbcL*-TN2, lower). Cells were grown in TAP medium under continuous light (∼30 μmol photons m^−2^ s^−1^) conditions until reaching the early logarithmic growth stage (∼1 × 10^6^ cells/mL). Cells were then collected and resuspended in TP medium. After 24 h of acclimation, cells were harvested for immunofluorescence observation. Representative immunofluorescence images were shown. Chlorophyll, chlorophyll autofluorescence, pinkish red; IF, indirect immunofluorescence, green; DAPI, staining of the nucleus and chloroplast DNA, blue; Merge, overlay of signals. Nucleus (N) and pyrenoid (P) are indicated with arrows. Scale bar, 5 μm. Note that the majority of cells from multiple fields of view under the microscope displayed consistent patterns of subcellular localization and similar results were obtained from two replicates.

### 3.6 Photosystem II activity and abundance of photosynthetic complexes

To compare the photosystem II (PSII) activity and stability among different strains, Chlamydomonas cells were cultured mixotrophically under constant white light (∼30 μmol photons m^−2^ s^−1^). In the absence of measurement light, values of the maximum quantum efficiency of PSII (ΦPSII, F_v_/F_m_) were less than 0.6 in both Δ*atpB* and Δ*rbcL* mutants (Fig. 5a–c), and those values of *atpB*-TNs and *rbcL*-TNs were similar to the chloroplast-complemented strains (∼0.75), indicating potential impairment of PSII in the mutants. As actinic light intensity increased, ΦPSII exhibited a rapid decline in the Δ*atpB* mutant, a much slower decline in *atpB*-C, and an intermediate trend in *atpB*-TNs, indicating that PSII is more unstable in the mutant and the stability is partially recovered in *atpB*-TNs (Fig. 5a). ΦPSII also demonstrated a rapid decline in the Δ*rbcL* mutant compared to *rbcL*-C; however, the stability is only slightly rescued in *rbcL*-TNs (Fig. 5c). Consistently, the reduction state of primary quinone acceptor Q_A_ (1-qL) for *atpB*-TNs was intermediate between Δ*atpB* mutant and *atpB*-C (Fig. 5b), whereas only slightly rescued in *rbcL*-TNs compared to Δ*rbcL* mutant and *rbcL*-C (Fig. 5d). A rapid upregulation of 1-qL represents accumulation of the reduced plastoquinone (PQ) pool and, consequently, a blockage in the downstream photosynthetic electron transport (PET) [37]. These results indicate that while a certain level of ATP synthase or Rubisco complexes can partially restore photosynthetic capacity in *atpB*-TNs and *rbcL*-TNs, downstream PET still remains constrained. This limitation is likely caused by inefficient consumption of the light-dependent-reaction products, i.e., H^+^, ATP, and NADPH, resulting in their accumulation and feedback inhibition of PET and PSII activities.

**Fig. 5:**
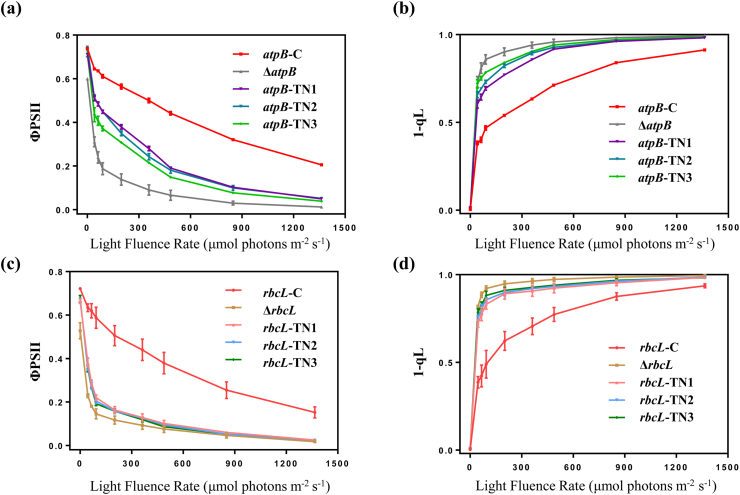
Chlorophyll fluorescence measurements of Chlamydomonas strains. Cells were cultured mixotrophically under ∼30 μmol photons m^−2^ s^−1^ white light to mid-log phase (∼3 × 10^6^ cells/mL). (a, c) Relative quantum yield of PSII (ΦPSII) in *atpB*-TNs (a) and *rbcL*-TNs (c) was measured on the light response curve setting. (b, d) Redox state of the PQ pool was measured by the 1-qL values of *atpB*-TNs (b) and *rbcL*-TNs (d) under increasing actinic light intensities. Each data point represents mean ± SD (n = 3).

To further evaluate the influence on photosynthetic components, we first compared the abundance of photosynthetic complexes by blue native polyacrylamide gel electrophoresis (BN-PAGE) analysis of the isolated thylakoid membranes. With equal amounts of chlorophyll loading, the Δ*atpB* mutant, *atpB*-TN2, and *atpB*-C showed comparable levels of photosystem supercomplexes, including PSII-LHCII and PSI-LHCI (Fig. 6a, left). Abundance of PSII dimer/ATP synthase, PSII/PSI monomer, and cytochrome *b*_6_*f* (Cyt *b*_6_*f*) was slightly reduced in the Δ*atpB* mutant but was restored in *atpB*-TN2 (Fig. 6a, left). We next analyzed photosynthesis-associated proteins using immunoblotting with equal protein loading. Results showed reduced accumulation of D1 and CP47 of PSII, and PetB of Cyt *b*_6_*f* in the Δ*atpB* mutant. This defect was largely rescued in *atpB*-TNs (Fig. 6b, left), consistent with the results in Fig. 6a (left). In contrast, the amount of PSI-LHCI supercomplex was remarkably reduced in the Δ*rbcL* mutant relative to *rbcL*-C (Fig. 6a, right). Immunoblotting analysis of denatured proteins demonstrated the identical results, e.g., accumulation of PSI-related proteins (including PsaA, LHCA3/6) was especially affected in the Δ*rbcL* mutant. This defect was partially (for PsaA and LHCA3; Fig. 6b, right) or fully (for LHCA6; Supplemental Fig. S4, right) rescued in *rbcL*-TNs.

**Fig. 6:**
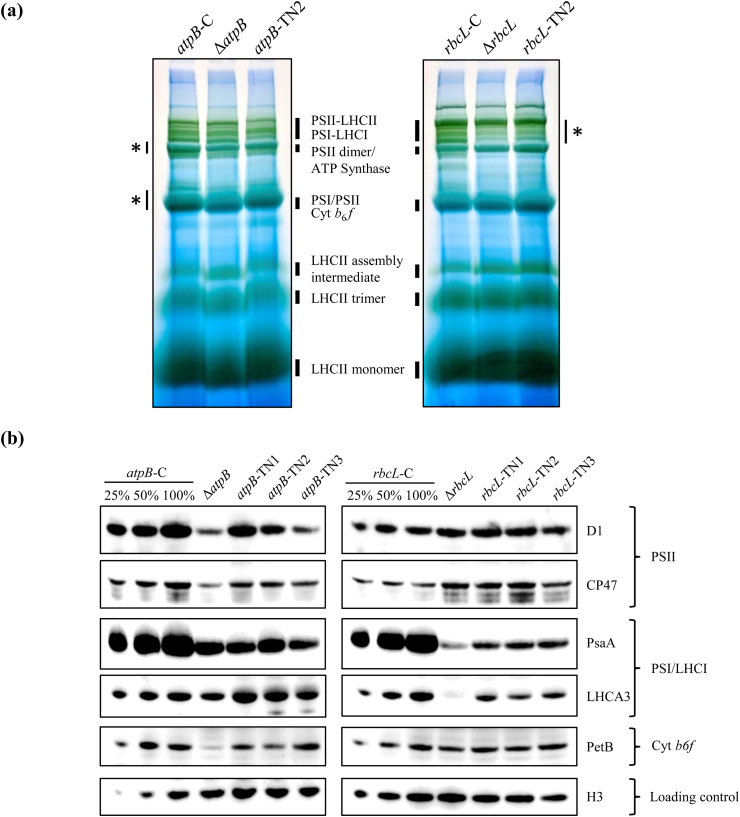
Accumulation of photosynthesis-associated protein complexes. (a) BN-PAGE analysis of photosynthesis-associated protein complexes. Cells were cultured in liquid TAP medium under continuous light illumination (∼30 μmol photons m^−2^ s^−1^) to the mid-log phase (∼3 × 10^6^ cells/mL). Thylakoid membranes were isolated and purified membranes were solubilized with 2% β-DM. Samples of equal chlorophyll content (∼20 μg) were loaded in each lane. The identities of the major complexes are indicated. Asterisks denote complexes with reduced abundance in the Δ*atpB* or Δ*rbcL* mutants compared to chloroplast-complemented strains *atpB*-C or *rbcL*-C. (b) Immunoblot analyses of photosystem-associated proteins. Each lane was loaded with ∼30 μg of total protein. A dilution series (25% and 50%) of *atpB*-C and *rbcL*-C was included for comparison. Histone H3 was used as an equal loading control. Similar results were obtained from two independent experiments.

## 4 Discussion

In this study, our analysis of chloroplast gene distribution shows that gene transfer to the nucleus is not equal among photosynthetic organisms derived from primary plastid endosymbiosis. We demonstrate that conserved genes retained in all analyzed chloroplast genomes of the selected 15 green lineage species, such as *atpB* and *rbcL*, can be transferred to the nucleus and partially substitute for their chloroplast copies, as Chlamydomonas strains with these two genes transferred to the nucleus exhibited partial rescue of photosynthetic capability. However, phenotypes of the two nuclear-transferred genes exhibited different levels of complementation: as *atpB*-TN strains exhibited considerable recovery, whereas *rbcL*-TN strains showed only minimal photoautotrophic growth under 5% CO_2_ but no growth in ambient air. This phenotypic distinction might be related to the protein expression levels, as ATPB and RBCL in the TN strains reached ∼10% and less than ∼6% of wild-type levels, respectively. Previous studies have established that photoautotrophic growth under ambient air requires at least 18% of wild-type Rubisco in tobacco [38] and 15% in Chlamydomonas [33]. By contrast, the ATPB threshold that supports photoautotrophic growth can be as low as 10% in tobacco [39]. These results suggest that *atpB* may be more amenable to nuclear transfer, while *rbcL* may require additional strategies to achieve a sufficient level of Rubisco accumulation for the photoautotrophic growth of Chlamydomonas cells.

Indeed, the *rbcL* gene has been reported to be amenable to relocation to the nucleus in tobacco, achieving ∼10% protein level and ∼3% activity of the wild-type Rubisco [40]. An *atpB rbcL* double gene mutant was constructed in maize using a TALE-cytidine deaminase, which showed a yellow phenotype that could be partially rescued by nuclear *atpB* expression [41]. However, the off-target effect of TALE may introduce unknown mutations in the transgenic lines, and the assessment of photosynthetic restoration is confounded by the presence of the additional *rbcL* mutation, limiting the ability to evaluate the contribution of *atpB* complementation. Here, we used single-gene-transfer transgenic lines to show that transferring either *atpB* or *rbcL* from the chloroplast to the nucleus can partially rescue growth defects of the respective Chlamydomonas mutants. We quantified the photosynthetic capacity to confirm the partial restoration of photosynthesis. The relatively low abundance of ATP synthase and Rubisco in TN strains may limit PET and result in partial restoration of photosynthetic complexes. These findings reveal a quantitative relationship between chloroplast protein accumulation and photosynthetic activity, laying a foundation for achieving complete physiological complementation via nuclear transfer in future studies.

Photosynthesis generates reactive oxygen species (ROS) within chloroplasts, leading to chronic oxidative damage to the chloroplast DNA. The transfer of chloroplast genes to the nuclear environment may serve as a strategy to protect these genes from ROS-induced damage, thereby promoting their stable inheritance [42]. Naturally occurring endosymbiotic gene transfer (EGT) is a slow process. It involves the integration of chloroplast DNA into the nuclear genome, the acquisition of promoter elements and cTP sequences, and the adaptive evolution of the codon usage to cater to nuclear expression. Rational design and selection of functional elements that are related to nuclear gene expression, protein synthesis and chloroplast relocalization, etc, can bypass the naturally occurring random evolutionary processes and accelerate chloroplast-to-nucleus gene transfer. However, although nucleus-encoded ATPB and RBCL can be relocated to chloroplast and assembled into holoenzyme complexes within the chloroplast, the achieved protein levels remain far lower than those of native chloroplast expression. One reasonable explanation lies in gene copy number. In contrast to the single-copy genes in the nuclear genome, chloroplast genes are present in hundreds of copies per cell due to the multiple genome copies within each chloroplast. This corresponds to the higher mRNA levels derived from chloroplast genomes compared to nuclear genes [43] and aligns with previous findings that chloroplast-based expression systems can typically produce heterologous proteins at much higher levels than nuclear-based systems [[44], [45], [46]].

Additionally, nuclear transformation is prone to positional effects. The chromosomal context of transgene integration site can result in epigenetic modifications, causing differential expression outcomes ranging from reduced accumulation to complete silencing [47,48]. Even with strategies known to counteract epigenetic silencing and boost nuclear gene expression [[49], [50], [51]], e.g., by using codon-optimized sequences, HSP70A/RBCS2 chimeric promoter, and intron insertion in the experimental design, protein levels of nuclear-expressed ATPB and RBCL were unable to reach those achieved with native chloroplast expression in *C. reinhardtii* (Fig. 3). In green lineage species, *RBCS* is encoded in the nuclear genome [52] and RBCS protein is one of the most abundant proteins in photosynthetic cells. The robust nuclear expression of *RBCS* gene is attributed to a complex regulatory mechanism [53,54] as well as the presence of a multi-copy gene family [55]. This implies that more sophisticated strategies, such as increasing copy numbers of nuclear genes, adaptive evolution, optimizing regulatory elements, and improving protein maturation and stability, etc, are required to dramatically enhance nuclear expression levels following gene transfer, ultimately enabling genes to reach functional equivalence with their chloroplast counterparts.

The chloroplast organelle contains approximately 3000 proteins, over 95% of which are encoded by the nuclear genome [5,6]. However, many photosynthesis-related core subunits are retained in the chloroplast genome (Supplemental Table S1), suggesting that selective constraints have prevented transfer of these genes to the nucleus. One such constraint may be related to the intrinsic properties of the proteins themselves. Many core subunits of photosynthetic complexes are hydrophobic transmembrane proteins. When encoded by the nucleus and synthesized in the cytosol, these hydrophobic proteins are prone to misfolding and aggregation in the aqueous cytoplasmic environment [56], posing challenges for their efficient targeting to the chloroplast. Furthermore, even if they successfully traverse the chloroplast envelope via TOC-TIC machinery, these proteins may require additional sorting and transport mechanisms, such as the chloroplast signal recognition particle (cpSRP) pathway [57] and the chloroplast twin arginine translocation (cpTat) pathway [58], for proper integration into the thylakoid membrane. Moreover, some proteins undergo co-translational integration into the thylakoid membrane to ensure proper assembly. For example, the D1 protein (encoded by PsbA) and CP47 (encoded by PsbB) are each co-translated with other proteins [59,60], with their nascent peptides being inserted into the thylakoid membrane during synthesis. This co-translational requirement may therefore explain why these genes are not already transferred to the nucleus in all extant photosynthetic species. Interestingly, overexpression of the D1 protein via nuclear transformation in tobacco, rice, and Arabidopsis has been shown to significantly enhance photosynthetic efficiency and plant biomass [61], indicating that the synthesized D1 protein in the cytosol might be retargeted to the chloroplast and assembled into functional PSII complex although no solid biochemical evidences were provided in this study. This raises the possibility that even the PsbA gene, and potentially other conserved photosynthetic genes encoding membrane proteins, might be amenable to functional transfer to the nucleus under specific conditions.

In summary, our work provides a foundational framework for the progressive transfer of chloroplast genes to the nucleus, with the ultimate goal of developing a minimal or engineered chloroplast genome in the model green alga *Chlamydomonas reinhardtii*. Many genes outside the conserved 41-gene set have undergone transfer to the nucleus in at least one photosynthetic species, suggesting that such genes may be amenable to transfer in *C. reinhardtii* as well. A minimal chloroplast gene set would not only define the core requirements for a photosynthetic life but would also shed light on investigating fundamental biological questions, i.e., the chloroplast-to-nucleus retrograde signaling under different types of environmental stresses, regulation of protein import into chloroplast and holoenzyme/supercomplex assembly. The principles established in this study may guide the redesign of the chloroplast as a photosynthetic factory with potential applications in enhancing photosynthetic efficiency and production of high-value bioproducts.

## Data availability statement

All relevant data are available from the corresponding author upon request.

## CRediT authorship contribution statement

**Weida Shi:** Conceptualization, Data curation, Formal analysis, Investigation, Methodology, Writing – original draft. **Mengyue Zhu:** Investigation, Methodology, Writing – original draft. **Chunhui Hou:** Investigation, Methodology. **Qiuling Fan:** Project administration, Resources, Supervision, Writing – review & editing. **Deqiang Duanmu:** Conceptualization, Formal analysis, Funding acquisition, Project administration, Resources, Supervision, Writing – original draft, Writing – review & editing.

## Declaration of competing interest

The authors declare that they have no known competing financial interests or personal relationships that could have appeared to influence the work reported in this paper.
