# Supplementary material for: Transfer of chloroplast rbcL and atpB genes to nucleus enables partial rescue of photoautotrophic growth of Chlamydomonas
Source: Synth Syst Biotechnol. 2026 Jul 30;17:14–25. doi: 10.1016/j.synbio.2026.07.004 (PMC13451854; doi:10.1016/j.synbio.2026.07.004)
Supplement: Supplementary file 1 [file mmc1.docx]

| Purpose | Primer name | Primer sequences | Annotation |
| --- | --- | --- | --- |
| Detection of WT- and TN- amplicons | cTP-s | cccatggccgcgctgaag | Sense primer targeting the cTP of RbcS2 |
|  | rbcL-TN-as | cacCTTGAAGCCGAACACGTTG | Antisense primer targeting the nuclear codon-optimized RbcL sequences |
|  | atpB-TN-as | GGTCTCCTCCACCTTCACGT | Antisense primer targeting the nuclear codon-optimized AtpB sequences |
|  | atpB-s | GTCAACCGCAGGGTAAATACCTT | Sense and antisense primers targeting the chloroplast atpB or rbcL coding sequences |
|  | atpB-as | GCTCTTGTATACGGTCAAATGAACG |  |
|  | rbcL-s | tcacaagtaccagcagtagcgt |  |
|  | rbcL-as | ggtgttccacctgaagaatgtgg |  |
| Verification of plasmid integration and homoplasmy test of the transgenes | F1 | CCAGCACCACCGAAAAGAC | Confirmation of atpB integration |
|  | R1 | CAATACACGTTCAAGTGCAGC |  |
|  | F2 | tgcgtggtatggaagttgtaga | Test of the homoplasmy of atpB transgene |
|  | R2 | accatgtgtgtattctaaataggt |  |
|  | F3 | GCTTTGAATCCAGCACCTGC | Confirmation of rbcL integration |
|  | R3 | CACGACCTAAGTATGCTTCACC |  |
|  | F4 | tggcacatcgtaatttataaagacagg | Test of the homoplasmy of rbcL transgene |
|  | R4 | ccggacagattattttaggatcgtc |  |

1. **Primers used in this study**
2. **Nuclear codon-optimized *atpB* sequence**

ATGCCCTGGGGCATCCTGATCCCCCTGACCATGAGCGACAGCATCGAGACCAAGAACATGGGCCGCATCGTGCAGATCATCGGCCCCGTGCTGGACATCGTGTTCGCCAAGGGCCAGGTGCCCAACATCTACAACGCCCTGACCATCCGCGCCAAGAACAGCGCCGGCACCGAGATGGCCGTGACCTGCGAGGTGCAGCAGCTGCTGGGCGACAACTGCGTGCGCGCCGTGAGCATGAACCCCACCGAGGGCCTGATGCGCGGCATGGAGGTGGTGGACACCGGCAAGCCCCTGAGCGTGCCCGTGGGCAAGGTGACCCTGGGCCGCATCTTCAACGTGCTGGGCGAGCCCGTGGACAACATGGgtgagtcgacgagcaagcccggcggatcaggcagcgtgcttgcagatttgacttgcaacgcccgcattgtgtcgacgaaggcttttggctcctctgtcgctgtctcaagcagcatctaaccctgcgtcgccgtttccatttgcagGCAACGTGAAGGTGGAGGAGACCCTGCCCATCCACCGCACCGCCCCCGCCTTCGTGGACCTGGACACCCGCCTGAGCATCTTCGAGACCGGCATCAAGGTGGTGGACCTGCTGGCCCCCTACCGCCGCGGCGGCAAGATCGGCCTGTTCGGCGGCGCCGGCGTGGGCAAGACCGTGCTGATCATGGAGCTGATCAACAACATCGCCAAGGCCCACGGCGGCGTGAGCGTGTTCGCCGGCGTGGGCGAGCGCACCCGCGAGGGCAACGACCTGTACACCGAGATGAAGGAGAGCGGCGTGATCGTGGAGAAGAACCTGAGCGACAGCAAGGTGGCCCTGGTGTACGGCCAGATGAACGAGCCCCCCGGCGCCCGCATGCGCGTGGCCCTGACCGCCCTGACCATGGCCGAGTACTTCCGCGACGTGAACAAGCAGGACGTGCTGTTCTTCATCGACAACATCTTCCGCTTCGTGCAGGCCGGCGCCGAGGTGAGCGCCCTGCTGGGCCGCATGCCCAGCGCCGTGGGCTACCAGCCCACCCTGGCCACCGAGATGGgtgagtcgacgagcaagcccggcggatcaggcagcgtgcttgcagatttgacttgcaacgcccgcattgtgtcgacgaaggcttttggctcctctgtcgctgtctcaagcagcatctaaccctgcgtcgccgtttccatttgcagGCGGCCTGCAGGAGCGCATCACCAGCACCAAGGACGGCAGCATCACCAGCATCCAGGCCGTGTACGTGCCCGCCGACGACCTGACCGACCCCGCCCCCGCCACCACCTTCGCCCACCTGGACGCCACCACCGTGCTGAGCCGCAACCTGGCCGCCAAGGGCATCTACCCCGCCGTGGACCCCCTGGAGAGCACCAGCACCATGCTGCAGCCCTGGATCCTGGGCGAGAAGCACTACGACAGCGCCCAGAGCGTGAAGAAGACCCTGCAGCGCTACAAGGAGCTGCAGGACATCATCGCCATCCTGGGCCTGGACGAGCTGAGCGAGGAGGACCGCCTGATCGTGGCCCGCGCCCGCAAGATCGAGCGCTTCCTGAGCCAGCCCTTCTTCGTGGCCGAGGTGTTCACCGGCAGCCCCGGCAAGTACGTGAGCCTGGCCGAGACCATCGAGGGCTTCGGCAAGATCTTCGCCGGCGAGCTGGACGACCTGCCCGAGCAGGCCTTCTACCTGGTGGGCAACATCACCGAGGCCATCAGCAAGGCCGCCAGCCTGAAGTAA

**(RbcS2 intron 1, marked in grey)**

3. **Nuclear codon-optimized *rbcL* sequence**

ATGGTGCCCCAGACCGAGACCAAGGCCGGCGCCGGCTTCAAGGCCGGCGTGAAGGACTACCGCCTGACCTACTACACCCCCGACTACGTGGTGCGCGACACCGACATCCTGGCCGCCTTCCGCATGACCCCCCAGCTGGGCGTGCCCCCCGAGGAGTGCGGCGCCGCCGTGGCCGCCGAGAGCAGCACCGGCACCTGGACCACCGTGTGGACCGACGGCCTGACCAGCCTGGACCGCTACAAGGGCCGCTGCTACGACATCGAGCCCGTGCCCGGCGAGGACAACCAGTACATCGCCTACGTGGCCTACCCCATCGACCTGTTCGAGGAGGGCAGCGTGACCAACATGTTCACCAGCATCGTGGGCAACGTGTTCGGCTTCAAGgtgagtcgacgagcaagcccggcggatcaggcagcgtgcttgcagatttgacttgcaacgcccgcattgtgtcgacgaaggcttttggctcctctgtcgctgtctcaagcagcatctaaccctgcgtcgccgtttccatttgcagGCCCTGCGCGCCCTGCGCCTGGAGGACCTGCGCATCCCCCCCGCCTACGTGAAGACCTTCGTGGGCCCCCCCCACGGCATCCAGGTGGAGCGCGACAAGCTGAACAAGTACGGCCGCGGCCTGCTGGGCTGCACCATCAAGCCCAAGCTGGGCCTGAGCGCCAAGAACTACGGCCGCGCCGTGTACGAGTGCCTGCGCGGCGGCCTGGACTTCACCAAGGACGACGAGAACGTGAACAGCCAGCCCTTCATGCGCTGGCGCGACCGCTTCCTGTTCGTGGCCGAGGCCATCTACAAGGCCCAGGCCGAGACCGGCGAGGTGAAGGGCCACTACCTGAACGCCACCGCCGGCACCTGCGAGGAGATGATGAAGCGCGCCGTGTGCGCCAAGGAGCTGGGCGTGCCCATCATCATGCACGACTACCTGACCGGCGGCTTCACCGCCAACACCAGCCTGGCCATCTACTGCCGCGACAACGGCCTGCTGCTGCACATCCACCGCGCCATGCACGCCGTGATCGACCGCCAGCGCAACCACGgtgagtcgacgagcaagcccggcggatcaggcagcgtgcttgcagatttgacttgcaacgcccgcattgtgtcgacgaaggcttttggctcctctgtcgctgtctcaagcagcatctaaccctgcgtcgccgtttccatttgcagGCATCCACTTCCGCGTGCTGGCCAAGGCCCTGCGCATGAGCGGCGGCGACCACCTGCACAGCGGCACCGTGGTGGGCAAGCTGGAGGGCGAGCGCGAGGTGACCCTGGGCTTCGTGGACCTGATGCGCGACGACTACGTGGAGAAGGACCGCAGCCGCGGCATCTACTTCACCCAGGACTGGTGCAGCATGCCCGGCGTGATGCCCGTGGCCAGCGGCGGCATCCACGTGTGGCACATGCCCGCCCTGGTGGAGATCTTCGGCGACGACGCCTGCCTGCAGTTCGGCGGCGGCACCCTGGGCCACCCCTGGGGCAACGCCCCCGGCGCCGCCGCCAACCGCGTGGCCCTGGAGGCCTGCACCCAGGCCCGCAACGAGGGCCGCGACCTGGCCCGCGAGGGCGGCGACGTGATCCGCAGCGCCTGCAAGTGGAGCCCCGAGCTGGCCGCCGCCTGCGAGGTGTGGAAGGAGATCAAGTTCGAGTTCGACACCATCGACAAGCTGTAA

**(RbcS2 intron 1, marked in grey)**

4. **Nuclear complementation plasmid pHyg3-ATPB**

Note: The construct contained the following elements: the HSP70A/RbcS2 promoter (positions 1–1158); the RbcS2 chloroplast transit peptide with RbcS2 intron 1 (positions 1165–1447); the nuclear codon‑optimized AtpB coding sequence with RbcS2 intron 1 (positions 1454–3219); the PsaD terminator (positions 3223–3839); the Aph7 expression unit which confers resistance against hygromycin B (positions 3846–5544); and the Amp expression unit (positions 6328–7188) which confers resistance against ampicillin.

Plasmid sequences:

acccaacgcccgaggtttcaccccgcctcacaccaaaccagcccgtcaaagcccttcaaaagctcacccaaagctcatagaaggcttccagtcgacagccatatcgccgccgctttggccacctccaaacagccccctccccgcaaagccgcacatgctgccggcggggcgtcacacaccagaccagaccagaccagcccgacattcaacacacacatggtctcatgcggtctgatggctttcctaagccaaccaggccggctcccagtgcagtgacgtgggcgtgacaggccgggtgctcccagccgcgtgccaattgccaaccccaccctacgcgaaggcattacgcgcctcaccgtgcattgctcctgctacagccccttgcaacaccgccgacctcgggaaggtggagttctcagcgcggtggccgcttgccccggccggcagctccgcagggcacacgtcacgcgaagggccgcgacggttcgagaaccgacttgagggcgccaaacgagcccgagccgccgttgcgccaggcgaaaccagaaccgtagattaatgcacttgagctattcattggagcgatctgccggggacagcgggtctggcgtgcgcgcgattggagatcgcaaattacatatgtctgcgtgacggcggggagctcgctgaggcttgacatgattggtgcgtatgtttgtatgaagctacaggactgatttggcgggctatgagggcgggggaagctctggaagggccgcgatggggcgcgcggcgtccagaaggcgccatacggcccgctggcggcacccatccggtataaaagcccgcgaccccgaacggtgacctccactttcagcgacaaacgagcacttatacatacgcgactattctgccgctatacataaccactcagctagcttaagatcccatcaagcttgcatgccgggcgcgccagaaggagcgcagccaaaccaggatgatgtttgatggggtatttgagcacttgcaacccttatccggaagccccctggcccacaaaggctaggcgccaatgcaagcagttcgcatgcagcccctggagcggtgccctcctgataaaccggccagggggcctatgttctttacttttttacaagagaagtcactcaacatcttaaaatgcatatggccgccgtcattgccaagtcctccgtctccgcggtgagtcgacgagcaagcccggcggatcaggcagcgtgcttgcagatttgacttgcaacgcccgcattgtgtcgacgaaggcttttggctcctctgtcgctgtctcaagcagcatctaaccctgcgtcgccgtttccatttgcaggccgtggcccgcccggcccgctccagcgtgcgccccatggccgcgctgaagcccgccgtcaaggccgcccccgtggctgccccggctcaggccaaccagatgaccggtATGCCCTGGGGCATCCTGATCCCCCTGACCATGAGCGACAGCATCGAGACCAAGAACATGGGCCGCATCGTGCAGATCATCGGCCCCGTGCTGGACATCGTGTTCGCCAAGGGCCAGGTGCCCAACATCTACAACGCCCTGACCATCCGCGCCAAGAACAGCGCCGGCACCGAGATGGCCGTGACCTGCGAGGTGCAGCAGCTGCTGGGCGACAACTGCGTGCGCGCCGTGAGCATGAACCCCACCGAGGGCCTGATGCGCGGCATGGAGGTGGTGGACACCGGCAAGCCCCTGAGCGTGCCCGTGGGCAAGGTGACCCTGGGCCGCATCTTCAACGTGCTGGGCGAGCCCGTGGACAACATGGgtgagtcgacgagcaagcccggcggatcaggcagcgtgcttgcagatttgacttgcaacgcccgcattgtgtcgacgaaggcttttggctcctctgtcgctgtctcaagcagcatctaaccctgcgtcgccgtttccatttgcagGCAACGTGAAGGTGGAGGAGACCCTGCCCATCCACCGCACCGCCCCCGCCTTCGTGGACCTGGACACCCGCCTGAGCATCTTCGAGACCGGCATCAAGGTGGTGGACCTGCTGGCCCCCTACCGCCGCGGCGGCAAGATCGGCCTGTTCGGCGGCGCCGGCGTGGGCAAGACCGTGCTGATCATGGAGCTGATCAACAACATCGCCAAGGCCCACGGCGGCGTGAGCGTGTTCGCCGGCGTGGGCGAGCGCACCCGCGAGGGCAACGACCTGTACACCGAGATGAAGGAGAGCGGCGTGATCGTGGAGAAGAACCTGAGCGACAGCAAGGTGGCCCTGGTGTACGGCCAGATGAACGAGCCCCCCGGCGCCCGCATGCGCGTGGCCCTGACCGCCCTGACCATGGCCGAGTACTTCCGCGACGTGAACAAGCAGGACGTGCTGTTCTTCATCGACAACATCTTCCGCTTCGTGCAGGCCGGCGCCGAGGTGAGCGCCCTGCTGGGCCGCATGCCCAGCGCCGTGGGCTACCAGCCCACCCTGGCCACCGAGATGGgtgagtcgacgagcaagcccggcggatcaggcagcgtgcttgcagatttgacttgcaacgcccgcattgtgtcgacgaaggcttttggctcctctgtcgctgtctcaagcagcatctaaccctgcgtcgccgtttccatttgcagGCGGCCTGCAGGAGCGCATCACCAGCACCAAGGACGGCAGCATCACCAGCATCCAGGCCGTGTACGTGCCCGCCGACGACCTGACCGACCCCGCCCCCGCCACCACCTTCGCCCACCTGGACGCCACCACCGTGCTGAGCCGCAACCTGGCCGCCAAGGGCATCTACCCCGCCGTGGACCCCCTGGAGAGCACCAGCACCATGCTGCAGCCCTGGATCCTGGGCGAGAAGCACTACGACAGCGCCCAGAGCGTGAAGAAGACCCTGCAGCGCTACAAGGAGCTGCAGGACATCATCGCCATCCTGGGCCTGGACGAGCTGAGCGAGGAGGACCGCCTGATCGTGGCCCGCGCCCGCAAGATCGAGCGCTTCCTGAGCCAGCCCTTCTTCGTGGCCGAGGTGTTCACCGGCAGCCCCGGCAAGTACGTGAGCCTGGCCGAGACCATCGAGGGCTTCGGCAAGATCTTCGCCGGCGAGCTGGACGACCTGCCCGAGCAGGCCTTCTACCTGGTGGGCAACATCACCGAGGCCATCAGCAAGGCCGCCAGCCTGAAGTAAgaattctggcagcagctggaccgcctgtaccatggagaagagctttacttgccgggatggccgatttcgctgattgatacgggatcggagctcggaggctttcgcgctaggggctaggcgaagggcagtggtgaccagggtcggtgtggggtcggcccacggtcaattagccacaggaggatcagggggaggtaggcacgtcgacttggtttgcgaccccgcagttttggcggacgtgctgttgtagatgttagcgtgtgcgtgagccagtggccaacgtgccacacccattgagaagaccaaccaacttactggcaatatctgccaatgccatactgcatgtaatggccaggccatgtgagagtttgccgtgcctgcgcgcgccccgggggcgggggggggacgggtggggggtagggggtctcacgggaacagcacgctaggggtcaggggggggggggggcgcagtttagctgaccagccgtgggatgatgcacgcatttgcaaggacagggtaatcacagcagcaacatggtgggcttaggacagctgtgggtcagtggacggacggcaggggagggacggcgcagctcgggagacagggggagacagcgtgactgaagcttctttcttgcgctatgacacttccagcaaaaggtagggcgggctgcgagacggcttcccggcgctgcatgcaacaccgatgatgcttcgaccccccgaagctccttcggggctgcatgggcgctccgatgccgctccagggcgagcgctgtttaaatagccaggcccccgattgcaaagacattatagcgagctaccaaagccatattcaaacacctagatcactaccacttctacacaggccactcgagcttgtgatcgcactccgctaagggggcgcctcttcctcttcgtttcagtcacaacccgcaaacatgacacaagaatccctgttacttctcgaccgtattgattcggatgattcctacgcgagcctgcggaacgaccaggaGttctgggaggtgagtcgacgagcaagcccggcggatcaggcagcgtgcttgcagatttgacttgcaacgcccgcattgtgtcgacgaaggcttttggctcctctgtcgctgtctcaagcagcatctaaccctgcgtcgccgtttccatttgcagccgctggcccgccgagccctggaggagctcgggctgccggtgccgccggtgctgcgggtgcccggcgagagcaccaaccccgtactggtcggcgagcccggcccggtgatcaagctgttcggcgagcactggtgcggtccggagagcctcgcgtcggagtcggaggcgtacgcggtcctggcggacgccccggtgccggtgccccgcctcctcggccgcggcgagctgcggcccggcaccggagcctggccgtggccctacctggtgatgagccggatgaccggcaccacctggcggtccgcgatggacggcacgaccgaccggaacgcgctgctcgccctggcccgcgaactcggccgggtgctcggccggctgcacagggtgccgctgaccgggaacaccgtgctcaccccccattccgaggtcttcccggaactgctgcgggaacgccgcgcggcgaccgtcgaggaccaccgcgggtggggctacctctcgccccggctgctggaccgcctggaggactggctgccggacgtggacacgctgctggccggccgcgaaccccggttcgtccacggcgacctgcacgggaccaacatcttcgtggacctggccgcgaccgaggtcaccgggatcgtcgacttcaccgacgtctatgcgggagactcccgctacagcctggtgcaactgcatctcaacgccttccggggcgaccgcgagatcctggccgcgctgctcgacggggcgcagtggaagcggaccgaggacttcgcccgcgaactgctcgccttcaccttcctgcacgacttcgaggtgttcgaggagaccccgctggatctctccggcttcaccgatccggaggaactggcgcagttcctctgggggccgccggacaccgcccccggcgcctgataaggatccccgctccgtgtaaatggaggcgctcgttgatctgagccttgccccctgacgaacggcggtggatggaagatactgctctcaagtgctgaagcggtagcttagctccccgtttcgtgctgatcagtctttttcaacacgtaaaaagcggaggagttttgcaattttgttggttgtaacgatcctccgttgattttggcctctttctccatgggcgggctgggcgtatttgaagcgggtacccaattcgccctatagtgagtcgtattacaattcactggccgtcgttttacaacgtcgtgactgggaaaaccctggcgttacccaacttaatcgccttgcagcacatccccctttcgccagctggcgtaatagcgaagaggcccgcaccgatcgcccttcccaacagttgcgcagcctgaatggcgaatggaaattgtaagcgttaatattttgttaaaattcgcgttaaatttttgttaaatcagctcattttttaaccaataggccgaaatcggcaaaatcccttataaatcaaaagaatagaccgagatagggttgagtgttgttccagtttggaacaagagtccactattaaagaacgtggactccaacgtcaaagggcgaaaaaccgtctatcagggcgatggcccactacgtgaaccatcaccctaatcaagttttttggggtcgaggtgccgtaaagcactaaatcggaaccctaaagggagcccccgatttagagcttgacggggaaagccggcgaacgtggcgagaaaggaagggaagaaagcgaaaggagcgggcgctagggcgctggcaagtgtagcggtcacgctgcgcgtaaccaccacacccgccgcgcttaatgcgccgctacagggcgcgtcaggtggcacttttcggggaaatgtgcgcggaacccctatttgtttatttttctaaatacattcaaatatgtatccgctcatgagacaataaccctgataaatgcttcaataatattgaaaaaggaagagtatgagtattcaacatttccgtgtcgcccttattcccttttttgcggcattttgccttcctgtttttgctcacccagaaacgctggtgaaagtaaaagatgctgaagatcagttgggtgcacgagtgggttacatcgaactggatctcaacagcggtaagatccttgagagttttcgccccgaagaacgttttccaatgatgagcacttttaaagttctgctatgtggcgcggtattatcccgtattgacgccgggcaagagcaactcggtcgccgcatacactattctcagaatgacttggttgagtactcaccagtcacagaaaagcatcttacggatggcatgacagtaagagaattatgcagtgctgccataaccatgagtgataacactgcggccaacttacttctgacaacgatcggaggaccgaaggagctaaccgcttttttgcacaacatgggggatcatgtaactcgccttgatcgttgggaaccggagctgaatgaagccataccaaacgacgagcgtgacaccacgatgcctgtagcaatggcaacaacgttgcgcaaactattaactggcgaactacttactctagcttcccggcaacaattaatagactggatggaggcggataaagttgcaggaccacttctgcgctcggcccttccggctggctggtttattgctgataaatctggagccggtgagcgtgggtctcgcggtatcattgcagcactggggccagatggtaagccctcccgtatcgtagttatctacacgacggggagtcaggcaactatggatgaacgaaatagacagatcgctgagataggtgcctcactgattaagcattggtaactgtcagaccaagtttactcatatatactttagattgatttaaaacttcatttttaatttaaaaggatctaggtgaagatcctttttgataatctcatgaccaaaatcccttaacgtgagttttcgttccactgagcgtcagaccccgtagaaaagatcaaaggatcttcttgagatcctttttttctgcgcgtaatctgctgcttgcaaacaaaaaaaccaccgctaccagcggtggtttgtttgccggatcaagagctaccaactctttttccgaaggtaactggcttcagcagagcgcagataccaaatactgtccttctagtgtagccgtagttaggccaccacttcaagaactctgtagcaccgcctacatacctcgctctgctaatcctgttaccagtggctgctgccagtggcgataagtcgtgtcttaccgggttggactcaagacgatagttaccggataaggcgcagcggtcgggctgaacggggggttcgtgcacacagcccagcttggagcgaacgacctacaccgaactgagatacctacagcgtgagctatgagaaagcgccacgcttcccgaagggagaaaggcggacaggtatccggtaagcggcagggtcggaacaggagagcgcacgagggagcttccagggggaaacgcctggtatctttatagtcctgtcgggtttcgccacctctgacttgagcgtcgatttttgtgatgctcgtcaggggggcggagcctatggaaaaacgccagcaacgcggcctttttacggttcctggccttttgctggccttttgctcacatgttctttcctgcgttatcccctgattctgtggataaccgtattaccgcctttgagtgagctgataccgctcgccgcagccgaacgaccgagcgcagcgagtcagtgagcgaggaagcggaagagcgcccaatacgcaaaccgcctctccccgcgcgttggccgattcattaatgcagctggcacgacaggtttcccgactggaaagcgggcagtgagcgcaacgcaattaatgtgagttagctcactcattaggcaccccaggctttacactttatgcttccggctcgtatgttgtgtggaattgtgagcggataacaatttcacacaggaaacagctatgaccatgattacgccaagctcgaaattaaccctcactaaagggaacaaaagctggagctccaccgcggtggcggccgctctagaacta

5. **Nuclear complementation plasmid pHyg3-RBCL**

Note: The construct contained the following elements: the HSP70A/RbcS2 promoter (positions 1–1158); the RbcS2 chloroplast transit peptide with RbcS2 intron 1 (positions 1165–1447); the nuclear codon‑optimized RbcL coding sequence with RbcS2 intron 1 (positions 1454–3171); the PsaD terminator (positions 3175–3791); the Aph7 expression unit which confers resistance against hygromycin B (positions 3798–5496); and the Amp expression unit (positions 6280–7140) which confers resistance against ampicillin.

Plasmid sequences:

acccaacgcccgaggtttcaccccgcctcacaccaaaccagcccgtcaaagcccttcaaaagctcacccaaagctcatagaaggcttccagtcgacagccatatcgccgccgctttggccacctccaaacagccccctccccgcaaagccgcacatgctgccggcggggcgtcacacaccagaccagaccagaccagcccgacattcaacacacacatggtctcatgcggtctgatggctttcctaagccaaccaggccggctcccagtgcagtgacgtgggcgtgacaggccgggtgctcccagccgcgtgccaattgccaaccccaccctacgcgaaggcattacgcgcctcaccgtgcattgctcctgctacagccccttgcaacaccgccgacctcgggaaggtggagttctcagcgcggtggccgcttgccccggccggcagctccgcagggcacacgtcacgcgaagggccgcgacggttcgagaaccgacttgagggcgccaaacgagcccgagccgccgttgcgccaggcgaaaccagaaccgtagattaatgcacttgagctattcattggagcgatctgccggggacagcgggtctggcgtgcgcgcgattggagatcgcaaattacatatgtctgcgtgacggcggggagctcgctgaggcttgacatgattggtgcgtatgtttgtatgaagctacaggactgatttggcgggctatgagggcgggggaagctctggaagggccgcgatggggcgcgcggcgtccagaaggcgccatacggcccgctggcggcacccatccggtataaaagcccgcgaccccgaacggtgacctccactttcagcgacaaacgagcacttatacatacgcgactattctgccgctatacataaccactcagctagcttaagatcccatcaagcttgcatgccgggcgcgccagaaggagcgcagccaaaccaggatgatgtttgatggggtatttgagcacttgcaacccttatccggaagccccctggcccacaaaggctaggcgccaatgcaagcagttcgcatgcagcccctggagcggtgccctcctgataaaccggccagggggcctatgttctttacttttttacaagagaagtcactcaacatcttaaaatgcatatggccgccgtcattgccaagtcctccgtctccgcggtgagtcgacgagcaagcccggcggatcaggcagcgtgcttgcagatttgacttgcaacgcccgcattgtgtcgacgaaggcttttggctcctctgtcgctgtctcaagcagcatctaaccctgcgtcgccgtttccatttgcaggccgtggcccgcccggcccgctccagcgtgcgccccatggccgcgctgaagcccgccgtcaaggccgcccccgtggctgccccggctcaggccaaccagatgaccggtATGGTGCCCCAGACCGAGACCAAGGCCGGCGCCGGCTTCAAGGCCGGCGTGAAGGACTACCGCCTGACCTACTACACCCCCGACTACGTGGTGCGCGACACCGACATCCTGGCCGCCTTCCGCATGACCCCCCAGCTGGGCGTGCCCCCCGAGGAGTGCGGCGCCGCCGTGGCCGCCGAGAGCAGCACCGGCACCTGGACCACCGTGTGGACCGACGGCCTGACCAGCCTGGACCGCTACAAGGGCCGCTGCTACGACATCGAGCCCGTGCCCGGCGAGGACAACCAGTACATCGCCTACGTGGCCTACCCCATCGACCTGTTCGAGGAGGGCAGCGTGACCAACATGTTCACCAGCATCGTGGGCAACGTGTTCGGCTTCAAGgtgagtcgacgagcaagcccggcggatcaggcagcgtgcttgcagatttgacttgcaacgcccgcattgtgtcgacgaaggcttttggctcctctgtcgctgtctcaagcagcatctaaccctgcgtcgccgtttccatttgcagGCCCTGCGCGCCCTGCGCCTGGAGGACCTGCGCATCCCCCCCGCCTACGTGAAGACCTTCGTGGGCCCCCCCCACGGCATCCAGGTGGAGCGCGACAAGCTGAACAAGTACGGCCGCGGCCTGCTGGGCTGCACCATCAAGCCCAAGCTGGGCCTGAGCGCCAAGAACTACGGCCGCGCCGTGTACGAGTGCCTGCGCGGCGGCCTGGACTTCACCAAGGACGACGAGAACGTGAACAGCCAGCCCTTCATGCGCTGGCGCGACCGCTTCCTGTTCGTGGCCGAGGCCATCTACAAGGCCCAGGCCGAGACCGGCGAGGTGAAGGGCCACTACCTGAACGCCACCGCCGGCACCTGCGAGGAGATGATGAAGCGCGCCGTGTGCGCCAAGGAGCTGGGCGTGCCCATCATCATGCACGACTACCTGACCGGCGGCTTCACCGCCAACACCAGCCTGGCCATCTACTGCCGCGACAACGGCCTGCTGCTGCACATCCACCGCGCCATGCACGCCGTGATCGACCGCCAGCGCAACCACGgtgagtcgacgagcaagcccggcggatcaggcagcgtgcttgcagatttgacttgcaacgcccgcattgtgtcgacgaaggcttttggctcctctgtcgctgtctcaagcagcatctaaccctgcgtcgccgtttccatttgcagGCATCCACTTCCGCGTGCTGGCCAAGGCCCTGCGCATGAGCGGCGGCGACCACCTGCACAGCGGCACCGTGGTGGGCAAGCTGGAGGGCGAGCGCGAGGTGACCCTGGGCTTCGTGGACCTGATGCGCGACGACTACGTGGAGAAGGACCGCAGCCGCGGCATCTACTTCACCCAGGACTGGTGCAGCATGCCCGGCGTGATGCCCGTGGCCAGCGGCGGCATCCACGTGTGGCACATGCCCGCCCTGGTGGAGATCTTCGGCGACGACGCCTGCCTGCAGTTCGGCGGCGGCACCCTGGGCCACCCCTGGGGCAACGCCCCCGGCGCCGCCGCCAACCGCGTGGCCCTGGAGGCCTGCACCCAGGCCCGCAACGAGGGCCGCGACCTGGCCCGCGAGGGCGGCGACGTGATCCGCAGCGCCTGCAAGTGGAGCCCCGAGCTGGCCGCCGCCTGCGAGGTGTGGAAGGAGATCAAGTTCGAGTTCGACACCATCGACAAGCTGTAAgaattctggcagcagctggaccgcctgtaccatggagaagagctttacttgccgggatggccgatttcgctgattgatacgggatcggagctcggaggctttcgcgctaggggctaggcgaagggcagtggtgaccagggtcggtgtggggtcggcccacggtcaattagccacaggaggatcagggggaggtaggcacgtcgacttggtttgcgaccccgcagttttggcggacgtgctgttgtagatgttagcgtgtgcgtgagccagtggccaacgtgccacacccattgagaagaccaaccaacttactggcaatatctgccaatgccatactgcatgtaatggccaggccatgtgagagtttgccgtgcctgcgcgcgccccgggggcgggggggggacgggtggggggtagggggtctcacgggaacagcacgctaggggtcaggggggggggggggcgcagtttagctgaccagccgtgggatgatgcacgcatttgcaaggacagggtaatcacagcagcaacatggtgggcttaggacagctgtgggtcagtggacggacggcaggggagggacggcgcagctcgggagacagggggagacagcgtgactgaagcttctttcttgcgctatgacacttccagcaaaaggtagggcgggctgcgagacggcttcccggcgctgcatgcaacaccgatgatgcttcgaccccccgaagctccttcggggctgcatgggcgctccgatgccgctccagggcgagcgctgtttaaatagccaggcccccgattgcaaagacattatagcgagctaccaaagccatattcaaacacctagatcactaccacttctacacaggccactcgagcttgtgatcgcactccgctaagggggcgcctcttcctcttcgtttcagtcacaacccgcaaacatgacacaagaatccctgttacttctcgaccgtattgattcggatgattcctacgcgagcctgcggaacgaccaggaGttctgggaggtgagtcgacgagcaagcccggcggatcaggcagcgtgcttgcagatttgacttgcaacgcccgcattgtgtcgacgaaggcttttggctcctctgtcgctgtctcaagcagcatctaaccctgcgtcgccgtttccatttgcagccgctggcccgccgagccctggaggagctcgggctgccggtgccgccggtgctgcgggtgcccggcgagagcaccaaccccgtactggtcggcgagcccggcccggtgatcaagctgttcggcgagcactggtgcggtccggagagcctcgcgtcggagtcggaggcgtacgcggtcctggcggacgccccggtgccggtgccccgcctcctcggccgcggcgagctgcggcccggcaccggagcctggccgtggccctacctggtgatgagccggatgaccggcaccacctggcggtccgcgatggacggcacgaccgaccggaacgcgctgctcgccctggcccgcgaactcggccgggtgctcggccggctgcacagggtgccgctgaccgggaacaccgtgctcaccccccattccgaggtcttcccggaactgctgcgggaacgccgcgcggcgaccgtcgaggaccaccgcgggtggggctacctctcgccccggctgctggaccgcctggaggactggctgccggacgtggacacgctgctggccggccgcgaaccccggttcgtccacggcgacctgcacgggaccaacatcttcgtggacctggccgcgaccgaggtcaccgggatcgtcgacttcaccgacgtctatgcgggagactcccgctacagcctggtgcaactgcatctcaacgccttccggggcgaccgcgagatcctggccgcgctgctcgacggggcgcagtggaagcggaccgaggacttcgcccgcgaactgctcgccttcaccttcctgcacgacttcgaggtgttcgaggagaccccgctggatctctccggcttcaccgatccggaggaactggcgcagttcctctgggggccgccggacaccgcccccggcgcctgataaggatccccgctccgtgtaaatggaggcgctcgttgatctgagccttgccccctgacgaacggcggtggatggaagatactgctctcaagtgctgaagcggtagcttagctccccgtttcgtgctgatcagtctttttcaacacgtaaaaagcggaggagttttgcaattttgttggttgtaacgatcctccgttgattttggcctctttctccatgggcgggctgggcgtatttgaagcgggtacccaattcgccctatagtgagtcgtattacaattcactggccgtcgttttacaacgtcgtgactgggaaaaccctggcgttacccaacttaatcgccttgcagcacatccccctttcgccagctggcgtaatagcgaagaggcccgcaccgatcgcccttcccaacagttgcgcagcctgaatggcgaatggaaattgtaagcgttaatattttgttaaaattcgcgttaaatttttgttaaatcagctcattttttaaccaataggccgaaatcggcaaaatcccttataaatcaaaagaatagaccgagatagggttgagtgttgttccagtttggaacaagagtccactattaaagaacgtggactccaacgtcaaagggcgaaaaaccgtctatcagggcgatggcccactacgtgaaccatcaccctaatcaagttttttggggtcgaggtgccgtaaagcactaaatcggaaccctaaagggagcccccgatttagagcttgacggggaaagccggcgaacgtggcgagaaaggaagggaagaaagcgaaaggagcgggcgctagggcgctggcaagtgtagcggtcacgctgcgcgtaaccaccacacccgccgcgcttaatgcgccgctacagggcgcgtcaggtggcacttttcggggaaatgtgcgcggaacccctatttgtttatttttctaaatacattcaaatatgtatccgctcatgagacaataaccctgataaatgcttcaataatattgaaaaaggaagagtatgagtattcaacatttccgtgtcgcccttattcccttttttgcggcattttgccttcctgtttttgctcacccagaaacgctggtgaaagtaaaagatgctgaagatcagttgggtgcacgagtgggttacatcgaactggatctcaacagcggtaagatccttgagagttttcgccccgaagaacgttttccaatgatgagcacttttaaagttctgctatgtggcgcggtattatcccgtattgacgccgggcaagagcaactcggtcgccgcatacactattctcagaatgacttggttgagtactcaccagtcacagaaaagcatcttacggatggcatgacagtaagagaattatgcagtgctgccataaccatgagtgataacactgcggccaacttacttctgacaacgatcggaggaccgaaggagctaaccgcttttttgcacaacatgggggatcatgtaactcgccttgatcgttgggaaccggagctgaatgaagccataccaaacgacgagcgtgacaccacgatgcctgtagcaatggcaacaacgttgcgcaaactattaactggcgaactacttactctagcttcccggcaacaattaatagactggatggaggcggataaagttgcaggaccacttctgcgctcggcccttccggctggctggtttattgctgataaatctggagccggtgagcgtgggtctcgcggtatcattgcagcactggggccagatggtaagccctcccgtatcgtagttatctacacgacggggagtcaggcaactatggatgaacgaaatagacagatcgctgagataggtgcctcactgattaagcattggtaactgtcagaccaagtttactcatatatactttagattgatttaaaacttcatttttaatttaaaaggatctaggtgaagatcctttttgataatctcatgaccaaaatcccttaacgtgagttttcgttccactgagcgtcagaccccgtagaaaagatcaaaggatcttcttgagatcctttttttctgcgcgtaatctgctgcttgcaaacaaaaaaaccaccgctaccagcggtggtttgtttgccggatcaagagctaccaactctttttccgaaggtaactggcttcagcagagcgcagataccaaatactgtccttctagtgtagccgtagttaggccaccacttcaagaactctgtagcaccgcctacatacctcgctctgctaatcctgttaccagtggctgctgccagtggcgataagtcgtgtcttaccgggttggactcaagacgatagttaccggataaggcgcagcggtcgggctgaacggggggttcgtgcacacagcccagcttggagcgaacgacctacaccgaactgagatacctacagcgtgagctatgagaaagcgccacgcttcccgaagggagaaaggcggacaggtatccggtaagcggcagggtcggaacaggagagcgcacgagggagcttccagggggaaacgcctggtatctttatagtcctgtcgggtttcgccacctctgacttgagcgtcgatttttgtgatgctcgtcaggggggcggagcctatggaaaaacgccagcaacgcggcctttttacggttcctggccttttgctggccttttgctcacatgttctttcctgcgttatcccctgattctgtggataaccgtattaccgcctttgagtgagctgataccgctcgccgcagccgaacgaccgagcgcagcgagtcagtgagcgaggaagcggaagagcgcccaatacgcaaaccgcctctccccgcgcgttggccgattcattaatgcagctggcacgacaggtttcccgactggaaagcgggcagtgagcgcaacgcaattaatgtgagttagctcactcattaggcaccccaggctttacactttatgcttccggctcgtatgttgtgtggaattgtgagcggataacaatttcacacaggaaacagctatgaccatgattacgccaagctcgaaattaaccctcactaaagggaacaaaagctggagctccaccgcggtggcggccgctctagaacta

6. **Chloroplast complementation plasmid pAtpB**

Detailed features of the plasmid include: the 5′ homologous arm (positions 185–1624); the 3′ homologous arm (positions 4179–5314); the region between the two arms corresponds to the sequence deleted in the Δ*atpB* mutant (positions 1454–3171); the *atpB* coding region (positions 3069–4544); and the ampicillin resistance cassette (positions 6534–7499).

Plasmid sequences:

gttaattaaaatttacatatttaatgctatttattattattgcaattgcattaaatatttttttaaaaaaaattaatcttcagctatattagtaaataacccataaatagtttcaattggaataattggaattggatatggactagttttattttcttctaataactttaatatcgctgctagcGATCCTCTGATGGAGTATCTCGCGATGCTCGATCTTGGTTTTTCTTAATTAGTAAACTATAGATAAATTTTAAAACAACCAATACTTCAGTTTGCTGTAGATAATTTACAGTACCAAATTCTAAATCTAAATCTTGTAAAAAGGTAATTAGAGTAACTTTTCTTTGGGGTAAAGATTTAGTATTTAACCAATTCAAGATACTAAGGTGAATAATAGATTGCTCACGAGTAGAACTGTTTACATATAGCTTAAACAAAAAAATAAGACCTTCTGTCGAATTTTTTAGATACAAGCAGTACTTATTTACTAAAAGTTTAAACTGGTTTTCTGTTTTTATATTCAAACCATTAAAATGTTCTACTAAACTGTCTTGTACGCTATAAATATCATTATTTGTCATAAATATAAATAAATGTAACTTTTGTTGTCGATCCTAAAATAATCTGTCCGGAAATATAATTTAAAAATCTTAAAAATTTTTAAATACATTTAAATATATTATATATTACTTTTTTTAAATATATTATATATTACTTTTTTTAAATATATTATATATTACTTTTTTTAAATATATTATATATTACTTTTTTTAAATATATTATATATTACTTTTTTTAAATATATTATATATTACTTTTTTTTAGTAAACTTTGACAAGCTTTACATCCCTACCCTACTTCCCAACAAATCTATCGGCAAAGCCGATTCCCAAAGGGGACAAGGAATGTAAATAGTCCATCGGCTTCCCCCATACCCTAATACCCTGAAAGGGTATATATCCCCGTAGGGGATGGAAGGGTATGATTGAGCGTCGTCCCCAAAGGGGAAGAAATTTGAGCTAGAGGATACGTCGGTCGCCTGAAAATAGTTGCTTCCCCAAAGGGGACAACCAAAGCTTGTCTTTTAAATGAAATGTCTCTTTCCCCAAAGGGGACCAGAGGAAAGCTTGTCTTTATGAGCCGTCCCCGAAGGGGTGAGGAAAGAAAGGGCAGTTGAAGAAATGTATTTTAGGACGTCCCCTTCCCCTTCCCCGAGAAAGAGTAAACTTAGGGATTTCAATCCGTAGTTAGGACGTCCTCCTTACGGAGTATTTAAAATCCCGGAGGGGATTAAATTTGTATGTTAATTTTTTGTATATTTTTAAGAATTTTATTATTATTATTATTATTATTATTATTATTATTATTATTATTATAAAACCGTATTACTATTCGTATTAgGTACCCCATTATATATTATACATTAAAAAAATAAAAAATTTTTTTTTATTTTATTTTTTTTATTTTATTAATTATTTTATTAATTATTTTATTAAATACCATGTGTGTATTCTAAATAGGTTTATTGGGGTACTTTATTTTACACTTTATTTTACACTTTATTTTACACTTTATTTTACACTTTATTTTACACTTTATTTTACACTTTATTTTACACTTTTTTAATATGAATTAAATTAAATTAATTTAATTTAATTTAAGAGAACCAATTACTGGTATAATTTGGAAAATAAAATACATTTAATCTGGTAATTTATTTGGTAATTTGGTCAAAATTATAATAGTTTTTATTTGATTTTTTTGAATTGAATTTAAAAAGACATCCCCTTACAGTATTCTTGCAAACCCTTACGGGACAATAAATAAATTTGTTGCCTGCCAACTGCCGATATTTATATACTAGGACGTCCCCTTCCCCTTACGGGATATTTATATACTCCGAAGGAGGCAAGTAAACTTAGGGATTTTAATGCAATAAATAAATTTGTCCCCTTACGGGAATATAAATATTAGTGGACGTCCCCTTCGGGCAAATAAATTTTAGTGGCAGTTGCCTCGCCTATCGGCTAACAAGTTCCTTCGGAGTATATAAATATAGGACGTCAGTGGCAGTTGCCTGCCAACTGCCTATATTTATATACTGCGATAAACTTTAGTTGCCCGAAGGGGTTTACATACTCCGAAGGAGGAAGCAGGCAGTGGCGGTACCACTGCCACTAAAATTTATTTGCCTCCTAACGGAGCATTAAAATCCCGAAGGGGACGTCCTGCCAACTGCCTGCTCCGCAGTATTAACATCCTATATTTATATACTCCGAAGTATATTTATATGCTTCCCCTTCCCCTTCGGGTATATAAATATTGGGCAAGTAAACTTAGGAGTATATAAATATAGGACGCCAGTGGCAGTGGTACCGCCACTGACGTCCACTAATATTTATATTCCCGTAAGGGGACGTCCTCCTTCCCCTTCCCCTTCGGGACGTCCCCTTCGGGTAAATAAATTTTAGTGGCAGTTGCCTGCCAACTGCCTAATATAAATATTGGGCAAGTAAACTTAGCATGTTTACATACTCCGAAGGAGGACGCCAGTGGCAGTGGTACAATAAATAAATTGTATGTAAACCTGCTACCGCAGCAAATAAATTTTATTCTATTTTAATACAATAAATAAATTTGTTGGCAGGCAACAAATTTATTTATTGTCCCGAAGGGGAAGGGGACGTCCTGAAGGGAAAGGTGCAACTACCTGGGAATTTTACCGATTAAATTAACCTTTTAAATAATTTTTTAAAAATACTTTTTTAATACCTTGTTTTGTTCTTAAAGAAACTGTATCTCGTGGACGACAAATATAATTTGGCGTTTTCACACTCTTATTATTTACTCGCACGTTTCCTTTAGTTTTTTGCTGAAAAAATAAAATTAGTATTTAACACATTATTAAATACACGTTTAACGGCGTCCTTTATTTTAAAGTAAATAGATTAAATAGCTAATATGACATATATAATGTATGTGTAATTAAAATAAATTGGCTCTTTAAGAAGAAAACAACTTAATGGTGTCCAAATATTTATTTTAATTACACATAAAAAATTAAATTATTTTAATGAAGCAGCTTTACTAATAGCTTCTGTAATGTTACCTACTAAGTAGAATGCTTGTTCTGGTAAATCATCTAATTCACCAGCGAAAATTTTACCAAAACCTTCAATAGTTTCAGCTAAAGAAACATATTTACCAGGTGAACCTGTAAATACTTCAGCTACGAAGAATGGTTGTGATAAGAAACGTTCAATTTTACGAGCACGAGCTACAATAAGTCTGTCTTCTTCAGATAATTCATCTAAACCTAGAATAGCAATAATATCTTGTAATTCTTTGTAACGTTGTAAAGTTTTTTTTACGCTTTGTGCAGAATCATAGTGTTTTTCACCAAGAATCCACGGTTGTAACATAGTTGAAGTTGATTCTAATGGGTCAACCGCAGGGTAAATACCTTTAGCAGCTAAGTTACGAGAAAGTACTGTAGTAGCATCTAAGTGAGCGAATGTTGTAGCAGGAGCAGGGTCAGTAAGGTCATctgcagGTACATATACAGCCTGAATCGATGTGATAGAACCATCTTTAGTTGAAGTAATACGTTCTTGAAGACCACCCATTTCTGTAGCTAAAGTAGGTTGGTAACCTACAGCTGATGGCATACGACCTAATAAAGCAGATACTTCAGCACCAGCTTGTACGAAACGGAAAATGTTATCAATGAAGAATAATACGTCTTGTTTGTTAACATCACGGAAATATTCAGCCATTGTTAAAGCTGTTAAAGCAACACGCATACGAGCACCTGGTGGTTCGTTCATTTGACCGTATACAAGAGCTACTTTTGAATCAGATAAGTTCTTTTCAACAATAACACCAGATTCTTTCATTTCTGTGTAAAGGTCGTTACCTTCACGTGTACGTTCACCAACACCAGCAAATACAGAAACACCACCGTGTGCTTTAGCAATGTTGTTGATCAGTTCCATAATTAAAACTGTTTTGCCTACACCAGCACCACCGAAAAGACCAATTTTACCACCACGACGGTATGGAGCTAAAAGGTCTACAACTTTAATACCTGTTTCAAAAATTGATAAACGAGTATCTAAATCAACGAAAGCAGGAGCTGTACGGTGAATTGGTAAAGTTTCTTCAACTTTAACATTACCCATGTTATCTACAGGTTCACCTAGAACGTTGAAAATACGTCCTAAAGTTACTTTACCTACAGGAACACTTAACGGTTTACCAGTATCTACAACTTCCATACCACGCATTAAACCTTCTGTTGGGTTCATAGAAACTGCACGTACACAGTTATCACCTAAAAGTTGTTGAACTTCACAAGTAACAGCCATTTCTGTACCTGCTGAGTTTTTAGCACGAATAGTTAGAGCGTTGTAAATATTTGGTACTTGGCCTTTAGCAAATACGATATCTAAAACAGGACCAATAATTTGTACAATACGTCCCATGTTTTTTGTTTCAATAGAATCACTCATAGTAAGTGGAATTAATATGCCCCAAGGCATTCATATTTTAACTTATTTTACTTAAATTCTTACGTATAAACCCCGAAGGAAACTTTATCTTAGGTAATTTGAAATAAGAACCTCCTCCTTCCCCTTCGGGCAAGTAAACTTAGCATGTTTACATACTCCGAAGGAGGACGCCAGTGGCAGTGGTATCGCCACTGCCTAGTATGTAAACATTGTGGCTTTACTTTCAAAAAGGCACCTTTAACTGAGAGTGTAACTTGTTATAAATTTAAAATTTATATAAAAGGAAGCTCAAACATATAGTAGAATTTATATATAAAAAGTATTATTCACTAACGCTTATTTTTTAGTTTTTTCATTTAACTATATATATTAATATATTATATAAATATTCCAAAACATTGTACTCATCTTTAGAGATGCAAAGCATCTTCTTTATTTATAAGCATACAATGTCTTGGAATATAAAATTAGTAAAAATATGGCATATTTTTAAATAGCTGGTGGAGAATTTTGAATTAAATTTTTATATTCTTGTACCACTTTAGTTAATTGTTGTGTTGAATAAGCTTGAGAAAGTTTCTTTTTCAATACTTTAATATTATGTTTTTCAATTAAATATTTACGTGGCATAATACCAACTGGTTGAACATTAATTTTTTGTTTTAAACTTGTTTTTTTAATATTGATTGATTTTAATTTTGGCATTTCAACAATTTCTAAACGTAAATAATCACCTGGCCATGTAAATCCACCGTTTTGTGGAATATAGCGTTGAAGAGGTTTTAAAACTTGTTGGAATTcgagctccagcttttgttccctttagtgagggttaatttcgagcttggcgtaatcatggtcatagctgtttcctgtgtgaaattgttatccgctcacaattccacacaacatacgagccggaagcataaagtgtaaagcctggggtgcctaatgagtgagctaactcacattaattgcgttgcgctcactgcccgctttccagtcgggaaacctgtcgtgccagctgcattaatgaatcggccaacgcgcggggagaggcggtttgcgtattgggcgctcttccgcttcctcgctcactgactcgctgcgctcggtcgttcggctgcggcgagcggtatcagctcactcaaaggcggtaatacggttatccacagaatcaggggataacgcaggaaagaacatgtgagcaaaaggccagcaaaaggccaggaaccgtaaaaaggccgcgttgctggcgtttttccataggctccgcccccctgacgagcatcacaaaaatcgacgctcaagtcagaggtggcgaaacccgacaggactataaagataccaggcgtttccccctggaagctccctcgtgcgctctcctgttccgaccctgccgcttaccggatacctgtccgcctttctcccttcgggaagcgtggcgctttctcatagctcacgctgtaggtatctcagttcggtgtaggtcgttcgctccaagctgggctgtgtgcacgaaccccccgttcagcccgaccgctgcgccttatccggtaactatcgtcttgagtccaacccggtaagacacgacttatcgccactggcagcagccactggtaacaggattagcagagcgaggtatgtaggcggtgctacagagttcttgaagtggtggcctaactacggctacactagaaggacagtatttggtatctgcgctctgctgaagccagttaccttcggaaaaagagttggtagctcttgatccggcaaacaaaccaccgctggtagcggtggtttttttgtttgcaagcagcagattacgcgcagaaaaaaaggatctcaagaagatcctttgatcttttctacggggtctgacgctcagtggaacgaaaactcacgttaagggattttggtcatgagattatcaaaaaggatcttcacctagatccttttaaattaaaaatgaagttttaaatcaatctaaagtatatatgagtaaacttggtctgacagttaccaatgcttaatcagtgaggcacctatctcagcgatctgtctatttcgttcatccatagttgcctgactccccgtcgtgtagataactacgatacgggagggcttaccatctggccccagtgctgcaatgataccgcgagacccacgctcaccggctccagatttatcagcaataaaccagccagccggaagggccgagcgcagaagtggtcctgcaactttatccgcctccatccagtctattaattgttgccgggaagctagagtaagtagttcgccagttaatagtttgcgcaacgttgttgccattgctacaggcatcgtggtgtcacgctcgtcgtttggtatggcttcattcagctccggttcccaacgatcaaggcgagttacatgatcccccatgttgtgcaaaaaagcggttagctccttcggtcctccgatcgttgtcagaagtaagttggccgcagtgttatcactcatggttatggcagcactgcataattctcttactgtcatgccatccgtaagatgcttttctgtgactggtgagtactcaaccaagtcattctgagaatagtgtatgcggcgaccgagttgctcttgcccggcgtcaatacgggataataccgcgccacatagcagaactttaaaagtgctcatcattggaaaacgttcttcggggcgaaaactctcaaggatcttaccgctgttgagatccagttcgatgtaacccactcgtgcacccaactgatcttcagcatcttttactttcaccagcgtttctgggtgagcaaaaacaggaaggcaaaatgccgcaaaaaagggaataagggcgacacggaaatgttgaatactcatactcttcctttttcaatattattgaagcatttatcagggttattgtctcatgagcggatacatatttgaatgtatttagaaaaataaacaaataggggttccgcgcacatttccccgaaaagtgccacctgacgcgccctgtagcggcgcattaagcgcggcgggtgtggtggttacgcgcagcgtgaccgctacacttgccagcgccctagcgcccgctcctttcgctttcttcccttcctttctcgccacgttcgccggctttccccgtcaagctctaaatcgggggctccctttagggttccgatttagtgctttacggcacctcgaccccaaaaaacttgattagggtgatggttcacgtagtgggccatcgccctgatagacggtttttcgccctttgacgttggagtccacgttctttaatagtggactcttgttccaaactggaacaacactcaaccctatctcggtctattcttttgatttataagggattttgccgatttcggcctattggttaaaaaatgagctgatttaacaaaaatttaacgcgaattttaacaaaatattaacgcttacaatttccattcgccattcaggctgcgcaactgttgggaagggcgatcggtgcgggcctcttcgctattacgccagctggcgaaagggggatgtgctgcaaggcgattaagttgggtaacgccagggttttcccagtcacgacgttgtaaaacgacggccagtgaattgtaatacgactcactatagggcgaattgggtaccgggccccccctcga

7. **Chloroplast complementation plasmid pRbcL**

Detailed features of the plasmid include: the 5′ homologous arm (positions 185–1320); the 3′ homologous arm (positions 2749–4099); the region between the two arms corresponds to the coding sequence of *rbcL* (positions 1321–2748), which is replaced by aadA in the Δ*rbcL* mutant; and the ampicillin resistance cassette (positions 5318–6178).

Plasmid sequences:

gttaattaaaatttacatatttaatgctatttattattattgcaattgcattaaatatttttttaaaaaaaattaatcttcagctatattagtaaataacccataaatagtttcaattggaataattggaattggatatggactagttttattttcttctaataactttaatatcgctgctagcaccagtacaacgaacacggctaccttcagtaatttttaaaccatcacctaataataccgcacctacgttgtttgcttctaagttaagtgcaatacctaaagtaccatcttcgaattcaagtaattcacctgacattgctttttctaaaccataaatacgagcaataccgtcacctacttggaaaacgataccgaaatctaccattttcacttctggagtgtattgttcaattaaatctttaataagattactaagttcttctggagtacgcattgccataaaaaagaaaaaataaataaaagattaaaaaagtttatttttaaaatctttctcgagaattttaaataagtttaaaattcaacaaaaatagtgagtggtaagatcacttgttaacaaaagtaatggttcacccttgtcatatttaaatactaaaattcatttgcccgaagaggacaaatttatttattgcattaaaatccctaagtttacttgcccgtaaggggaagggggggacgtccacaggcgtcgtaagcaactaaagtttatgacgccgattgctttgttaggaaaatataaatatcccataagaaaaggtcctttaaaggttttatggactaaataaaaaagatagcataagcattaaaatcatgcaaattaaaaaaaaggtaaatgtatttataaaaaggtaaatgtatttatatagtatttatattatagcataataataaatatatttataaattgattgttcttagagctaaaagagaagaacaatgggtttataggtattttgagatccagttataaaaatgacttttgacgtttatggtatataaacactgcctctaataaagtctactatattggagaggagtgaacagtggcctcgcttatcccctgacaggaatatacatggttttagtaagtaaactgcgtaagacgaccgacatatacctaaaggccctttctatgctcgactgataagacaagtacataaatttgctagtttacattattttttatttctaaatatataatatatttaaatgtatttaaaatttttcaacaatttttaaattatatttccggacagattattttaggatcgtcaaaagaagttacatttatttatataaatggttccacaaacagaaactaaagcaggtgctggattcaaagccggtgtaaaagactaccgtttaacatactacacacctgattacgtagtaagagatactgatattttagctgcattccgtatgactccacaaccaggtgttccacctgaagaatgtggtgctgctgtagctgctgaatcttcaacaggtacatggactacagtatggactgacggtttaacaagtcttgaccgttacaaaggtcgttgttacgatatcgaaccagttccgggtgaagacaaccaatacattgcttacgtagcttacccaatcgacttattcgaagaaggttcagtaactaacatgttcacttctattgtaggtaacgtattcggtttcaaagctttacgtgctctacgtcttgaagaccttcgtattccacctgcttacgttaaaacattcgtaggtcctccacacggtattcaggtagaacgtgacaaattaaacaaatatggtcgtggtcttttaggttgtacaatcaaacctaaattaggtctttcagctaaaaactacggtcgtgcagtttatgaatgtttacgtggtggtcttgactttactaaagacgacgaaaacgtaaactcacaaccattcatgcgttggcgtgaccgtttccttttcgttgctgaagctatttacaaagctcaagcagaaacaggtgaagttaaaggtcactacttaaacgctactgctggtacttgtgaagaaatgatgaaacgtgcagtatgtgctaaagaattaggtgtacctattattatgcacgactacttaacaggtggtttcacagctaacacttcattagctatctactgtcgtgacaacggtcttcttctacacatccaccgtgctatgcacgcggttattgaccgtcaacgtaaccacggtattcacttccgtgttcttgctaaagctcttcgtatgtctggtggtgaccaccttcactctggtactgttgtaggtaaactagaaggtgaacgtgaagttactctaggtttcgtagacttaatgcgtgatgactacgttgaaaaagaccgtagccgtggtatttacttcactcaagactggtgttcaatgccaggtgttatgccagttgcttcaggtggtattcacgtatggcacatgccagctttagttgaaatcttcggtgatgacgcatgtcttcagttcggtggtggtactctaggtcacccttggggtaacgctccaggtgctgcagctaaccgtgtagctcttgaagcttgtactcaagctcgtaacgaaggtcgtgaccttgctcgtgaaggtggcgacgtaattcgttcagcttgtaaatggtctccagaacttgctgctgcatgtgaagtttggaaagaaattaaattcgaatttgatactattgacaaactttaatttttatttttcatgatgtttatgtgaatagcataaacatcgtttttatttttatggtgtttaggttaaatacctaaacatcattttacatttttaaaattaagttctaaagttatcttttgtttaaatttgcctgtctttataaattacgatgtgccagaaaaataaaatcttagctttttattatagaatttatctttatgtattatattttataagtTATaataaaagaaatagtaacatactaaagcggatgtaactcaatcggtagagtgcgatccttccaagttcgaggttgtgggttcgagtcccatcatccgctaaaccaatctataaaagttgttgaatatgctgaaatgttttcaaagaaaaagcctagtttttcttttacaacaagcaaagaacaattggcattctttgattgtaagaaaatgcgcttacgtggagttatgtgtttttacttaagtgttttttaagaaccacagtgggaataacttgttaactgtatcatctgtaatataaatatatatctaaagacttcattagtagtgaaataaggttttttacgcattataaaatcggttagacatttatattactaaatgtctaaccgatttaaaagtaccacatcgggtcgcacgtatatgctgatctgatgaggtagtaaataaagtacattacattaaccgaaacgacctgaagtcgaagcaattaagaatgctgcgtaagtaaagatataacctactgagaagtgagctaaacctactaaacgagcttgtacaatagaaagagcaactggtttgtctttccagtatactaagttagctagaggtgttttttcgtgagcccaaacaagagtttcaattaactcttgccagtaaccacgccaagaaattaagaacatgaaaccagtagcgtaaattaagtgaccaaataagaaagtccaagcccaaacagataatgagttcataccaaatgggttgtaaccattaattaattgagaagagtttaaccataggtagtcacgtaaccaacccattaagtaagtagacgattcatcgaattgtgctacgttaccttgccataaagttaaatgtttccagtgccagtagaatgttacccaaccaatagtgttaagcatccagaatacagctaagtagaaagcatcgtaagctgaaatgtcacaagtaccgccacgaccaggaccgtcacatgggaagctgtaaccaaagtcttttttatctggcattagtttagaaccacgagcatcaagagcacctttaacaaggattaatgttgtagtgtgaagacctaaagcaatagcgtggtgaacaaggaagtcaccagggccgagctccagcttttgttccctttagtgagggttaatttcgagcttggcgtaatcatggtcatagctgtttcctgtgtgaaattgttatccgctcacaattccacacaacatacgagccggaagcataaagtgtaaagcctggggtgcctaatgagtgagctaactcacattaattgcgttgcgctcactgcccgctttccagtcgggaaacctgtcgtgccagctgcattaatgaatcggccaacgcgcggggagaggcggtttgcgtattgggcgctcttccgcttcctcgctcactgactcgctgcgctcggtcgttcggctgcggcgagcggtatcagctcactcaaaggcggtaatacggttatccacagaatcaggggataacgcaggaaagaacatgtgagcaaaaggccagcaaaaggccaggaaccgtaaaaaggccgcgttgctggcgtttttccataggctccgcccccctgacgagcatcacaaaaatcgacgctcaagtcagaggtggcgaaacccgacaggactataaagataccaggcgtttccccctggaagctccctcgtgcgctctcctgttccgaccctgccgcttaccggatacctgtccgcctttctcccttcgggaagcgtggcgctttctcatagctcacgctgtaggtatctcagttcggtgtaggtcgttcgctccaagctgggctgtgtgcacgaaccccccgttcagcccgaccgctgcgccttatccggtaactatcgtcttgagtccaacccggtaagacacgacttatcgccactggcagcagccactggtaacaggattagcagagcgaggtatgtaggcggtgctacagagttcttgaagtggtggcctaactacggctacactagaaggacagtatttggtatctgcgctctgctgaagccagttaccttcggaaaaagagttggtagctcttgatccggcaaacaaaccaccgctggtagcggtggtttttttgtttgcaagcagcagattacgcgcagaaaaaaaggatctcaagaagatcctttgatcttttctacggggtctgacgctcagtggaacgaaaactcacgttaagggattttggtcatgagattatcaaaaaggatcttcacctagatccttttaaattaaaaatgaagttttaaatcaatctaaagtatatatgagtaaacttggtctgacagttaccaatgcttaatcagtgaggcacctatctcagcgatctgtctatttcgttcatccatagttgcctgactccccgtcgtgtagataactacgatacgggagggcttaccatctggccccagtgctgcaatgataccgcgagacccacgctcaccggctccagatttatcagcaataaaccagccagccggaagggccgagcgcagaagtggtcctgcaactttatccgcctccatccagtctattaattgttgccgggaagctagagtaagtagttcgccagttaatagtttgcgcaacgttgttgccattgctacaggcatcgtggtgtcacgctcgtcgtttggtatggcttcattcagctccggttcccaacgatcaaggcgagttacatgatcccccatgttgtgcaaaaaagcggttagctccttcggtcctccgatcgttgtcagaagtaagttggccgcagtgttatcactcatggttatggcagcactgcataattctcttactgtcatgccatccgtaagatgcttttctgtgactggtgagtactcaaccaagtcattctgagaatagtgtatgcggcgaccgagttgctcttgcccggcgtcaatacgggataataccgcgccacatagcagaactttaaaagtgctcatcattggaaaacgttcttcggggcgaaaactctcaaggatcttaccgctgttgagatccagttcgatgtaacccactcgtgcacccaactgatcttcagcatcttttactttcaccagcgtttctgggtgagcaaaaacaggaaggcaaaatgccgcaaaaaagggaataagggcgacacggaaatgttgaatactcatactcttcctttttcaatattattgaagcatttatcagggttattgtctcatgagcggatacatatttgaatgtatttagaaaaataaacaaataggggttccgcgcacatttccccgaaaagtgccacctgacgcgccctgtagcggcgcattaagcgcggcgggtgtggtggttacgcgcagcgtgaccgctacacttgccagcgccctagcgcccgctcctttcgctttcttcccttcctttctcgccacgttcgccggctttccccgtcaagctctaaatcgggggctccctttagggttccgatttagtgctttacggcacctcgaccccaaaaaacttgattagggtgatggttcacgtagtgggccatcgccctgatagacggtttttcgccctttgacgttggagtccacgttctttaatagtggactcttgttccaaactggaacaacactcaaccctatctcggtctattcttttgatttataagggattttgccgatttcggcctattggttaaaaaatgagctgatttaacaaaaatttaacgcgaattttaacaaaatattaacgcttacaatttccattcgccattcaggctgcgcaactgttgggaagggcgatcggtgcgggcctcttcgctattacgccagctggcgaaagggggatgtgctgcaaggcgattaagttgggtaacgccagggttttcccagtcacgacgttgtaaaacgacggccagtgaattgtaatacgactcactatagggcgaattgggtaccgggccccccctcga
